# Supplementary material for: Accuracy of information on the underlying cause of death: An analysis in Colombia during the COVID-19 pandemic in 2021
Source: PLoS One. 2025 May 20;20(5):e0320466. doi: 10.1371/journal.pone.0320466 (PMC12092014; doi:10.1371/journal.pone.0320466)
Supplement: S1 File — (DOCX) [file pone.0320466.s001.docx]

**Telephone interview guide to obtain missing information in the reviewed medical records**

Interview date.

Department/District:

Municipality:

The interviewer:

- . The researcher identifies themselves as representatives of the National Institute of Health, explains the study’s purpose, and outlines the protocol.

Ask the selected interviewee:

- What was your relationship with the deceased person?

- Do you authorize answering the interview?

If yes, obtain verbal informed consent.

- Do you authorize the audio recording of the call?

If not, ask permission to take written notes.

In relation to the deceased:

- What happened during the illness or situation that led to death?

- What do you think the person died from?

- Thank the interviewee for their participation.

The interviewer states the concept of the possible cause of death (CD) for the deceased person.

**Guía de entrevista telefónica para obtener la información faltante en las historias clínicas revisadas**

Fecha de la entrevista

Departamento/Distrito

Municipio

El entrevistador:

- Se identifica

- Explica el objetivo del proyecto

Pregunta al entrevistado seleccionado:

- ¿Cuál era su parentesco o relación con la persona fallecida?
- ¿Autoriza responder la entrevista?

En caso de ser afirmativo, obtiene el consentimiento informado verbal.

- ¿Autoriza la grabación en audio de la llamada?

En caso negativo pide permiso para tomar notas escritas.

En relación con el fallecido:

- ¿Qué sucedió durante la enfermedad o situación que llevó a la muerte?
- ¿De qué cree usted que murió la persona?
- Agradece la participación al entrevistado

El entrevistador (a) consigna el concepto sobre la posible causa de muerte de la persona fallecida
