# Supplementary material for: Accuracy of information on the underlying cause of death: An analysis in Colombia during the COVID-19 pandemic in 2021
Source: PLoS One. 2025 May 20;20(5):e0320466. doi: 10.1371/journal.pone.0320466 (PMC12092014; doi:10.1371/journal.pone.0320466)
Supplement: S1 Table — (DOCX) [file pone.0320466.s003.docx]

Table 1S. Underlying CoD agreement metrics between original and gold standard death certificates according to ICD-10 mortality list 2. Colombia, 2021

| **ICD-10 List 2** | **Death numbers** | | **Match between certificates** | **False positive %** | **False negative %** | **Kappa** | **SE** | **Kappa CI 95%** | |
| --- | --- | --- | --- | --- | --- | --- | --- | --- | --- |
|  | **Original** | **Standard** |  |  |  |  |  | **LL** | **UL** |
| 2 | 0 | 3 | 0 | - | 0,39 | 0,00 | 0,00 | 0,00 | 0,00 |
| 3 | 0 | 1 | 0 | - | 0,13 | 0,00 | 0,00 | 0,00 | 0,00 |
| 4 | 5 | 3 | 3 | 40,00 | 0,00 | 0,75 | 0,17 | 0,41 | 1,09 |
| 11 | 5 | 0 | 0 | 100,00 | 0,00 | 0,00 | 0,00 | 0,00 | 0,00 |
| 16 | 0 | 2 | 0 | - | 0,26 | 0,00 | 0,00 | 0,00 | 0,00 |
| 18 | 0 | 0 | 0 | - | 0,00 | - | - | - | - |
| 19 | 9 | 9 | 7 | 22,22 | 0,26 | 0,78 | 0,11 | 0,56 | 0,99 |
| 22 | 2 | 2 | 2 | 0,00 | 0,00 | 1,00 | 0,00 | 1,00 | 1,00 |
| 24 | 3 | 2 | 1 | 66,67 | 0,13 | 0,40 | 0,28 | -0,15 | 0,94 |
| 25 | 6 | 5 | 5 | 16,67 | 0,00 | 0,91 | 0,09 | 0,73 | 1,09 |
| 26 | 3 | 2 | 2 | 33,33 | 0,00 | 0,80 | 0,20 | 0,41 | 1,18 |
| 27 | 15 | 11 | 11 | 26,67 | 0,00 | 0,84 | 0,08 | 0,69 | 0,99 |
| 28 | 9 | 11 | 8 | 11,11 | 0,39 | 0,80 | 0,10 | 0,60 | 0,99 |
| 29 | 5 | 7 | 4 | 20,00 | 0,39 | 0,66 | 0,16 | 0,35 | 0,97 |
| 30 | 4 | 2 | 2 | 50,00 | 0,00 | 0,67 | 0,22 | 0,23 | 1,10 |
| 31 | 0 | 1 | 0 | 100,00 | 0,00 | 0,00 | 0,00 | 0,00 | 0,00 |
| 32 | 11 | 10 | 6 | 45,45 | 0,52 | 0,57 | 0,13 | 0,31 | 0,82 |
| 33 | 1 | 0 | 0 | 100,00 | 0,00 | 0,00 | 0,00 | 0,00 | 0,00 |
| 34 | 9 | 10 | 8 | 11,11 | 0,26 | 0,84 | 0,09 | 0,66 | 1,02 |
| 35 | 9 | 8 | 7 | 22,22 | 0,13 | 0,82 | 0,10 | 0,62 | 1,02 |
| 37 | 3 | 3 | 3 | 0,00 | 0,00 | 1,00 | 0,00 | 1,00 | 1,00 |
| 38 | 9 | 10 | 6 | 33,33 | 0,52 | 0,63 | 0,13 | 0,37 | 0,88 |
| 39 | 1 | 0 | 0 | 100,00 | 0,00 | 0,00 | 0,00 | 0,00 | 0,00 |
| 40 | 2 | 4 | 2 | 0,00 | 0,26 | 0,67 | 0,22 | 0,23 | 1,10 |
| 41 | 2 | 2 | 1 | 50,00 | 0,13 | 0,50 | 0,31 | -0,10 | 1,10 |
| 43 | 4 | 9 | 4 | 0,00 | 0,65 | 0,61 | 0,16 | 0,30 | 0,92 |
| 44 | 21 | 23 | 11 | 47,62 | 1,59 | 0,49 | 0,09 | 0,30 | 0,67 |
| 45 | 2 | 2 | 1 | 50,00 | 0,13 | 0,50 | 0,31 | -0,10 | 1,10 |
| 46 | 28 | 19 | 15 | 46,43 | 0,53 | 0,63 | 0,08 | 0,46 | 0,79 |
| 47 | 9 | 4 | 1 | 88,89 | 0,39 | 0,15 | 0,14 | -0,12 | 0,41 |
| 49 | 2 | 2 | 2 | 0,00 | 0,00 | 1,00 | 0,00 | 1,00 | 1,00 |
| 50 | 4 | 4 | 2 | 50,00 | 0,26 | 0,50 | 0,22 | 0,07 | 0,92 |
| 51 | 24 | 1 | 0 | 100,00 | 0,13 | 0,00 | 0,00 | -0,01 | 0,00 |
| 52 | 0 | 27 | 0 | - | 3,48 | 0,00 | 0,00 | 0,00 | 0,00 |
| 53 | 102 | 79 | 50 | 50,98 | 4,30 | 0,49 | 0,05 | 0,40 | 0,59 |
| 54 | 13 | 16 | 4 | 69,23 | 1,57 | 0,26 | 0,11 | 0,05 | 0,48 |
| 55 | 37 | 46 | 25 | 32,43 | 2,84 | 0,58 | 0,07 | 0,45 | 0,71 |
| 56 | 1 | 0 | 0 | 100,00 | 0,00 | 0,00 | 0,00 | 0,00 | 0,00 |
| 57 | 8 | 3 | 2 | 75,00 | 0,13 | 0,36 | 0,19 | 0,00 | 0,72 |
| 59 | 25 | 20 | 5 | 80,00 | 2,00 | 0,20 | 0,08 | 0,03 | 0,36 |
| 61 | 24 | 26 | 15 | 37,50 | 0,66 | 0,59 | 0,08 | 0,42 | 0,75 |
| 62 | 8 | 6 | 0 | 100,00 | 0,78 | -0,01 | 0,00 | -0,01 | 0,00 |
| 63 | 6 | 1 | 1 | 83,33 | 0,00 | 0,28 | 0,22 | -0,15 | 0,72 |
| 64 | 13 | 10 | 8 | 38,46 | 0,26 | 0,69 | 0,11 | 0,47 | 0,91 |
| 65 | 0 | 3 | 0 | - | 0,39 | 0,00 | 0,00 | 0,00 | 0,00 |
| 69 | 7 | 10 | 6 | 14,29 | 0,52 | 0,70 | 0,13 | 0,45 | 0,95 |
| 70 | 9 | 9 | 6 | 33,33 | 0,39 | 0,66 | 0,13 | 0,41 | 0,92 |
| 71 | 7 | 5 | 0 | 100 | 0,65 | -0,01 | 0,00 | -0,01 | 0,00 |
| 72 | 82 | 76 | 44 | 42,11 | 5,43 | 0,51 | 0,05 | 0,41 | 0,61 |
| 74 | 0 | 3 | 0 | - | 0,39 | 0,00 | 0,00 | 0,00 | 0,00 |
| 76 | 0 | 1 | 0 | - | 0,13 | 0,00 | 0,00 | 0,00 | 0,00 |
| 80 | 2 | 9 | 0 | 100,00 | 1,16 | 0,00 | 0,00 | -0,01 | 0,00 |
| Covid 19 | 231 | 247 | 212 | 8,23 | 6,42 | 0,84 | 0,02 | 0,80 | 0,88 |

SE: standard error LL: lower limit UL: upper limit
