# Supplementary material for: Accuracy of information on the underlying cause of death: An analysis in Colombia during the COVID-19 pandemic in 2021
Source: PLoS One. 2025 May 20;20(5):e0320466. doi: 10.1371/journal.pone.0320466 (PMC12092014; doi:10.1371/journal.pone.0320466)
Supplement: Attachment — Submitted filename: PONE-D-23-33548_R1_reviewer.pdf [file pone.0320466.s007.pdf]

PLOS ONE

# Accuracy of Information on the Underlying Cause of Death: An Analysis in Colombia during the COVID-19 Pandemic in 2021

--Manuscript Draft--

| | |
|---|---|
| Manuscript Number: | PONE-D-23-33548R1 |
| Article Type: | Research Article |
| Full Title: | Accuracy of Information on the Underlying Cause of Death: An Analysis in Colombia during the COVID-19 Pandemic in 2021 |
| Short Title: | Accuracy of information on the underlying cause of death in Colombia |
| Corresponding Author: | Laura Berrio-Parra, M.D<br>Instituto Nacional de Salud<br>Bogotá, Bogotá D.C COLOMBIA |
| Keywords: | Underlying cause of death; Death certificate; Medical information; Data accuracy; Colombia. |
| Abstract: | Objective: This study aimed to estimate the accuracy of the underlying Cause of Death (CoD) in the original death certificate, compared with a gold standard certificate based on information from clinical charts and relatives, in population deceased in Colombia during 2021.<br>Methods: A sample size of 806 deaths across 92 municipalities in Colombia were estimated from the pool of 326,833 original certificates provided by the National Department of Statistics. A two-stage stratified random sample with replacement was employed for selection. Information from medical records of the deceased and, when necessary, interviews with relatives or witnesses were used to determine CoDs on the gold standard certificate. We analyzed and compared the underlying CoD of the original and standard death certificates to estimate the level of accuracy. Measures of concordance, patterns of false positives and negatives, and calculate a kappa value were utilized as metrics to evaluate the quality of the death certificates.<br>Results:Information was obtained from 776, representing 96% of the desired sample. The concordance between original and gold standard certificates, categorized according to the ICD-10 chapters, was found to be 74%. Higher levels of agreement were observed for “codes for special COVID-19 situations” (kappa=0.84) and neoplasms (kappa=0.84). Overestimation was identified for "circulatory system diseases" (Chapter IX);(Chapter IX); "pregnancy, childbirth and puerperium" (Chapter XV); "signs, symptoms, and poorly defined conditions" (Chapter XVIII) and "diseases of the respiratory system" (Chapter X), while underestimation in "diseases of the genitourinary system" (Chapter XIV) among CoD.<br>Conclusions: The level of concordance between the original and gold standard death certificates was deemed adequate. However, improvements in the death certification process in Colombia are recommended, emphasizing the enhancement of training programs for health professionals. |
| Order of Authors: | Pablo Chaparro-Narváez, MD, PhD Public Health |
| | Jessika Alexandra Manrique Sanchez, MD, Esp demography, Esp Epidemiology |
| | Laura Berrio-Parra, MD,MSc Public Health, Specialist Epidemiology |
| | Diana Carolina Urrego Ricaurte, MD, MSc Public Health |
| | Luis José Torres Rojas, MD, Esp Epidemiology |
| | Nidia Patricia Orjuela Cantor, MSc Public Health |
| | Carlos Castañeda-Orjuela, MD, PhD Public Health, MSc Economy, ESP Biostatist |
| Response to Reviewers: | We gratefully received the revisor comments on our manuscript. Therefore, we made several adjustments to the manuscript addressing all aspects for its publication. In the response letter to the reviewers, we sent all the issues in which there were doubts, comments or suggestions. We uploaded a response letter to the reviewers, the revised manuscript with tracked changes, the manuscript, a figure, ethics committee approval, and supporting information files with all relevant data..We hope that the adjustments |

Powered by Editorial Manager® and ProduXion Manager® from Aries Systems Corporation

| | |
|---|---|
| | have responded to the questions and comments sent by the reviewers and that the uploaded files are in accordance with the requirements of Plos One. We are awaiting for the new revision! |
| **Additional Information:** | |
| **Question** | **Response** |
| **Financial Disclosure**<br><br>Enter a financial disclosure statement that describes the sources of funding for the work included in this submission. Review the submission guidelines for detailed requirements. View published research articles from *PLOS ONE* for specific examples.<br><br>This statement is required for submission and **will appear in the published article** if the submission is accepted. Please make sure it is accurate.<br><br>**Funded studies**<br>Enter a statement with the following details:<br>• Initials of the authors who received each award<br>• Grant numbers awarded to each author<br>• The full name of each funder<br>• URL of each funder website<br>• Did the sponsors or funders play any role in the study design, data collection and analysis, decision to publish, or preparation of the manuscript?<br><br>Did you receive funding for this work? | Yes |
| Please add funding details.<br>as follow-up to "**Financial Disclosure**<br><br>Enter a financial disclosure statement that describes the sources of funding for the work included in this submission. Review the submission guidelines for detailed requirements. View published research articles from *PLOS ONE* for specific examples.<br><br>This statement is required for submission and **will appear in the published article** if the submission is accepted. Please make | The CDC funded the research, providing financial support for the researchers and to do the field work necessary to collect the information. URL: https://www.cdc.gov/index.htm<br><br>The CDC did not pay a salary directly to the researchers. The financing was made to the public budget of the National Institute of Health.<br><br>This study does not have grant numbers per author because it is not a Grant.<br><br>"The funders had no role in study design, data collection and analysis, decision to publish, or preparation of the manuscript." |

| | |
|---|---|
| sure it is accurate.<br><br>**Funded studies**<br>Enter a statement with the following details:<br>• Initials of the authors who received each award<br>• Grant numbers awarded to each author<br>• The full name of each funder<br>• URL of each funder website<br>• Did the sponsors or funders play any role in the study design, data collection and analysis, decision to publish, or preparation of the manuscript?<br><br>Did you receive funding for this work?" | |
| Please select the country of your main research funder (please select carefully as in some cases this is used in fee calculation).<br>as follow-up to "**Financial Disclosure**<br><br>Enter a financial disclosure statement that describes the sources of funding for the work included in this submission. Review the submission guidelines for detailed requirements. View published research articles from *PLOS ONE* for specific examples.<br><br>This statement is required for submission and **will appear in the published article** if the submission is accepted. Please make sure it is accurate.<br><br>**Funded studies**<br>Enter a statement with the following details:<br>• Initials of the authors who received each award<br>• Grant numbers awarded to each author<br>• The full name of each funder<br>• URL of each funder website<br>• Did the sponsors or funders play any role in the study design, data collection and analysis, decision to publish, or preparation of the manuscript?<br><br>Did you receive funding for this work?" | UNITED STATES - US |

Powered by Editorial Manager® and ProduXion Manager® from Aries Systems Corporation

| | |
|---|---|
| **Competing Interests**<br><br>Use the instructions below to enter a competing interest statement for this submission. On behalf of all authors, disclose any competing interests that could be perceived to bias this work—acknowledging all financial support and any other relevant financial or non-financial competing interests.<br><br>This statement is required for submission and **will appear in the published article** if the submission is accepted. Please make sure it is accurate and that any funding sources listed in your Funding Information later in the submission form are also declared in your Financial Disclosure statement.<br><br>View published research articles from *PLOS ONE* for specific examples.<br><br>**NO authors have competing interests**<br><br>Enter: *The authors have declared that no competing interests exist.*<br><br>**Authors with competing interests**<br><br>Enter competing interest details beginning with this statement:<br><br>*I have read the journal's policy and the authors of this manuscript have the following competing interests: [insert competing interests here]*<br><br>* typeset | None of the authors have any conflicts of interest. |
| **Ethics Statement**<br><br>Enter an ethics statement for this submission. This statement is required if the study involved:<br><br>• Human participants<br>• Human specimens or tissue<br>• Vertebrate animals or cephalopods | The project received approval from the Committee on Ethics and Research Methodologies of the National Institute of Health (In Spanish Comité de Ética y Metodologías de la Investigación - CEMIN). Access to anonymized mortality databases from DANE was granted, and the extraction of information from the medical records by health professionals was conducted without accessing the CoDs from the original certificate to prevent potential bias. CEMIN also authorized the review of medical records in the capacity of the public health authority of the National Institute of Health, and these documents remained securely under the custody of the research team and the respective territorial entities. Telephone interviews, conducted with prior verbal |

| | |
|---|---|
| • Vertebrate embryos or tissues<br>• Field research<br><br>Write "N/A" if the submission does not require an ethics statement.<br><br>General guidance is provided below. Consult the submission guidelines for detailed instructions. **Make sure that all information entered here is included in the Methods section of the manuscript.** | consent, were carried out as required. During these interviews, the nature of the project, the information sought, and its intended use were clearly explained to the participants. The voluntary nature of their participation was emphasized, and the data analyzed was rigorously anonymized. This procedure was endorsed by CEMIN, ensuring ethical standards and the protection of participants' confidentiality throughout the research process.<br><br>The approval of the ethics committee was attached in Spanish and the translation of the same was attached in another file. |

Powered by Editorial Manager® and ProduXion Manager® from Aries Systems Corporation

| | |
|---|---|
| **Format for specific study types**<br><br>**Human Subject Research (involving human participants and/or tissue)**<br>• Give the name of the institutional review board or ethics committee that approved the study<br>• Include the approval number and/or a statement indicating approval of this research<br>• Indicate the form of consent obtained (written/oral) or the reason that consent was not obtained (e.g. the data were analyzed anonymously)<br><br>**Animal Research (involving vertebrate animals, embryos or tissues)**<br>• Provide the name of the Institutional Animal Care and Use Committee (IACUC) or other relevant ethics board that reviewed the study protocol, and indicate whether they approved this research or granted a formal waiver of ethical approval<br>• Include an approval number if one was obtained<br>• If the study involved *non-human primates*, add *additional details* about animal welfare and steps taken to ameliorate suffering<br>• If anesthesia, euthanasia, or any kind of animal sacrifice is part of the study, include briefly which substances and/or methods were applied<br><br>**Field Research**<br><br>Include the following details if this study involves the collection of plant, animal, or other materials from a natural setting:<br>• Field permit number<br>• Name of the institution or relevant body that granted permission | |
| **Data Availability**<br><br>Authors are required to make all data underlying the findings described fully available, without restriction, and from the time of publication. PLOS allows rare exceptions to address legal and ethical concerns. See the PLOS Data Policy and FAQ for detailed information. | Yes - all data are fully available without restriction |

| | |
|---|---|
| A Data Availability Statement describing where the data can be found is required at submission. Your answers to this question constitute the Data Availability Statement and **will be published in the article**, if accepted.<br><br>**Important:** Stating 'data available on request from the author' is not sufficient. If your data are only available upon request, select 'No' for the first question and explain your exceptional situation in the text box.<br><br>Do the authors confirm that all data underlying the findings described in their manuscript are fully available without restriction? | |
| **Describe where the data may be found in full sentences. If you are copying our sample text, replace any instances of XXX with the appropriate details.**<br><br>• If the data are **held or will be held in a public repository**, include URLs, accession numbers or DOIs. If this information will only be available after acceptance, indicate this by ticking the box below. For example: *All XXX files are available from the XXX database (accession number(s) XXX, XXX.).*<br>• If the data are all contained **within the manuscript and/or Supporting Information files**, enter the following: *All relevant data are within the manuscript and its Supporting Information files.*<br>• If neither of these applies but you are able to provide **details of access elsewhere**, with or without limitations, please do so. For example:<br><br>*Data cannot be shared publicly because of [XXX]. Data are available from the XXX Institutional Data Access / Ethics Committee (contact via XXX) for researchers who meet the criteria for access to confidential data.*<br><br>*The data underlying the results presented in the study are available from (include the name of the third party* | All relevant data are in the manuscript and its supporting information files. |

| | |
|---|---|
| *and contact information or URL).*<br>• This text is appropriate if the data are owned by a third party and authors do not have permission to share the data.<br><br>* typeset | |
| Additional data availability information: | Tick here if your circumstances are not covered by the questions above and you need the journal’s help to make your data available. |

Manuscript Click here to access/download;Manuscript;manuscript.docx

# Accuracy of information on the underlying cause of death: an analysis in Colombia during the COVID-19 pandemic in 2021

Pablo Chaparro-Narváez[1¶], Jessika Alexandra Manrique Sanchez[1¶], Laura Berrio-Parra[1¶*], Diana Carolina Urrego Ricaurte[1¶], Luis José Torres-Rojas[1¶], Nidia Patricia Orjuela Cantor[1¶], Carlos Castañeda-Orjuela[1¶]

**Affiliations**

[1] National Health Observatory, National Institute of Health, Bogotá, Colombia.

*lberrio@ins.gov.co (LB)

// Abstract

**Objective:** This study aimed to estimate the accuracy of the underlying Cause of Death (CoD) in the original death certificate, compared with a gold standard certificate based on information from clinical charts and relatives, in population deceased in Colombia during 2021.

**Methods:** A sample size of 806 deaths across 92 municipalities in Colombia were estimated from the pool of 326,833 original certificates provided by the National Department of Statistics. A two-stage stratified random sample with replacement was employed for selection. Information from medical records of the deceased and, when necessary, interviews with relatives or witnesses were used to determine CoDs on the gold standard certificate. We analyzed and compared the underlying CoD of the original and standard death certificates to estimate the level of accuracy. Measures of concordance, patterns of false positives and negatives, and calculate a kappa value were utilized as metrics to evaluate the quality of the death certificates.

**Results:** Information was obtained from 776, representing 96% of the desired sample. The concordance between original and gold standard certificates, categorized according to the ICD-10 chapters, was found to be 74%. Higher levels of agreement were observed for “codes for special COVID-19 situations” (kappa=0.84) and neoplasms (kappa=0.84). Overestimation was identified for "circulatory system diseases" (Chapter IX); "pregnancy, childbirth and puerperium" (Chapter XV); "signs, symptoms, and poorly defined conditions" (Chapter XVIII) and "diseases of the respiratory system" (Chapter X), while underestimation in "diseases of the genitourinary system" (Chapter XIV) among CoD.

**Conclusions:** The level of concordance between the original and gold standard death certificates was deemed adequate. However, improvements in the death certification

process in Colombia are recommended, emphasizing the enhancement of training programs for health professionals.

***Keywords:*** *Underlying cause of death; Death certificate; Medical information; Data accuracy; Colombia.*

# Introduction

The global impact of the COVID-19 pandemic underscored the vulnerabilities inherent in Civil Registration and Vital Statistics (CRVS) systems worldwide, emphasizing the imperative to enhance information systems (1). Effective public policy formulation relies on comprehensive, detailed, continuous, and real-time statistics related to population health and mortality, which are integral components provided by the CRVS system (2).

Encompassing vital events such as births, deaths, and others, the CRVS system not only yields legal and administrative advantages for individuals and families but also supplies crucial statistical or administrative information for policymakers. Continuous registration of deaths, along with their causes, serves as a cornerstone for informed policy formulation aimed at reducing premature mortality. In systems with inherent weaknesses, the response to health threats may be delayed, resulting in a higher incidence of deaths. Conversely, timely mortality statistics enable surveillance, considering the evolving trend in deaths burden for any cause, providing essential up-to-date data for informed decision-making (3).

The information on the Cause of Death (CoD) plays a pivotal role in guiding decision-making process, influencing policy direction, research funding priorities, and evaluating the effectiveness of public health interventions (4,5). Seven Sustainable Development Goals (SDGs) specifically rely on mortality data sourced from CRVS systems (6). However, the accuracy and timeliness of data recorded on death certificates can exhibit significant

variability (7–9). Deficiencies in CoD information quality can stem from factor such as inadequate training among coders, unfamiliarity with causes (7,10), time constraints (6), and a lack of supervision, leading to the frequent use of imprecise “garbage codes” (11). In addition to these factors the implementation of effective procedures to consolidate the information and the adherence to statistical standards that transform individual codes into comprehensive national mortality statistics play a crucial role (8). Notably, errors in CoD reporting are prevalent, with rates ranges between 39% and 61% in population-based studies and 32% to 45% in hospital studies (7–9).

Concerning the reporting landscape in the Americas in 2019, underreporting was estimated at 4%, accompanied by the utilization of poorly defined or absent CoD codes (3%) and the inclusion of garbage codes (15%). Conversely, Colombia exhibited higher underreporting at 13%, with poorly defined and ignored CoD accounting for 1%, and garbage codes for 11% (12). Despite these statistics, Colombia’s studies on CoD have been limited, focusing on aspects such as the knowledge of different CRVS actors (13), the quality of certification in general and cancer mortality (14–16), the certification quality of live births and non-fetal deaths in hospitals (15,17), and the quality of information for children under one year of age (18). In light of this, our study aims to fill this research gap by estimating the accuracy of underlying CoD in the original death certificate, compared with a gold standard certificate based on information from clinical charts and relatives, in population deceased in Colombia during 2021.

# Methods

A cross-sectional descriptive study was conducted in Colombia during in 2021. At the study’s commencement, the National Department on Statistics (DANE, by the Spanish acronym of *Departamento Administrativo Nacional de Estadística*) had compiled 326,833 non-fetal

death certificates, excluding those related to violent causes certified by the Institute of Legal Medicine and Forensic Sciences, as well as municipalities without reported COVID-19 infections or deaths. The selection criteria for the study included municipalities classified as large and intermediate, where cases of COVID-19 were identified based to the complexity of health care services; and the presence of more than 20 deaths associated with COVID-19, as determined in medical records or death certificate.

A two-stage stratified random sample with replacement was designed. The first stage considered conglomerates comprising 33 territorial entities (32 departments and the district of Bogotá). In the second stage, 92 municipalities were selected, with the population stratified by age groups (<60 years and 60 and more years). The estimated sample size was 806 deaths across the 92 municipalities, with a confidence level of 95%, an expected proportion of 70%, an estimation precision of 5%, and an effect of 2.5 for the two-stage sampling design.

Information from the medical records was collected and analyzed by a team of health professionals, including five doctors and a nurse with a postgraduate degree in epidemiology and public health. The professional's underwent prior training in the DANE vital statistics course to ensure accurate assignment of CoDs in the gold standard certificate. The review of medical records took place between 01/06/2022 and 30/09/2022.

The identification data of the deceased, as present in the original death certificate, were used to request corresponding medical records from territorial health entities. The health professionals, under strict supervision, extracted information from these medical records to complete the underlying CoD in the standard death certificate, considered the gold standard certificate for this study. To mitigate bias, health professionals never had access to the original death certificate. In cases where the medical records lacked sufficient information

to establish the CoD, interview with relatives, acquaintances, or witnesses were interviewed conducted.

An expert nosologist coded the CoD assigned by the health personnel, selecting the underlying CoD based on the International Classification of Diseases, 10$^{th}$ Revision (ICD-10) norms used in Colombia`s official mortality statistics. There was no CoD adjudicating committee; instead, the CoDs were directly collected from the medical records by the health professionals.

The gold standard certificate, based on detailed medical records, was deemed the most accurate source. A comparison of the CoD on original and gold standard certificates was conducted to identify false positives, false negatives, and kappa value, along with their respective 95% confidence interval (95% CI). False positive and false negative percentages were used to quantify the degree of overdiagnosis and under-certification, respectively. Kappa values greater than 0.81 were considered almost perfect, 0.61 to 0.80 as high, 0.41 to 0.60 as moderate, 0.21 to 0.40 as acceptable, 0.00 to 0.20 as low, and those < 0.00 as poor (19). The change in cause-specific mortality fraction (CSMF) was also calculated (20) as follows:

$$\mathrm{C}SMF = \frac{CaSMF^{S} - CaSMF^{\circ}}{CaSMF^{\circ}}\ x\,100$$

Where $CaSMF$ = cause specific mortality fraction, O = “original” certificate, S = “standard” certificate.

$$CaSMF = \frac{Number\ of\ deaths\ due\ to\ one\ specific\ cause}{Total\ number\ of\ deaths}$$

Additionally, to assess variations in the underlying CoDs between original and gold standard certificates the Bland-Altman diagram was employed to visualize the comparison. The collected information was processed in Microsoft Excel spreadsheets and analyzed using Stata, version 12.0.

The project received approval from the Committee on Ethics and Research Methodologies of the National Institute of Health (In Spanish *Comité de Ética y Metodologías de la Investigación* - CEMIN). Access to anonymized mortality databases from DANE was granted, and the extraction of information from the medical records by health professionals was conducted without accessing the CoDs from the original certificate to prevent potential bias. CEMIN also authorized the review of medical records in the capacity of the public health authority of the National Institute of Health, and these documents remained securely under the custody of the research team and the respective territorial entities. Telephone interviews, conducted with prior verbal consent, were carried out as required. During these interviews, the nature of the project, the information sought, and its intended use were clearly explained to the participants. The voluntary nature of their participation was emphasized, and the data analyzed was rigorously anonymized. This procedure was endorsed by CEMIN, ensuring ethical standards and the protection of participants' confidentiality throughout the research process.

# Results

In our sample of 776 deaths, stratification was conducted by department, municipality, sex and age, ensuring a representative cross-section of Colombia`s total mortality. Despite a nominal 4% loss in the sample, this minimal reduction did not introduce any distortion to the study's findings. The mean age of death was 65 years (range: 0-98 years; median: 68 years). Most deaths occurred in urban areas (94.2%) and hospitals (78.5%). CoD determination primarily relied on medical records (93.9%), with a 1% of the sample drawing from a multifaceted array of sources such as medical records, laboratory tests and interview with relatives or witnesses (Table 1). Overall, the agreement on the underlying CoD, based on ICD-10, was robust at 74% (575 deaths).

**Table 1. Characteristics of analyzed deaths. Colombia, 2021**

| | Women (n = 352) | | Men (n = 424) | | Total (n = 776) | |
|---|---|---|---|---|---|---|
| | n | % | n | % | n | % |
| Age | | | | | | |
| Mean age (SD) at death, in years | 65.9 | 21.4 | 64.4 | 20.9 | 65.1 | 21.1 |
| 0 to 9 | 12 | 3.4 | 15 | 3.5 | 27 | 3.5 |
| 10 to 19 | 2 | 0.6 | 6 | 1,4 | 8 | 1.0 |
| 20 to 29 | 10 | 2.8 | 6 | 1.4 | 16 | 2.1 |
| 30 to 39 | 15 | 4.3 | 21 | 5.0 | 36 | 4.6 |
| 40 to 49 | 22 | 6.3 | 31 | 7.3 | 53 | 6.8 |
| 50 to 59 | 42 | 11.9 | 62 | 14.6 | 104 | 13.4 |
| 60 to 69 | 80 | 22.7 | 83 | 19.6 | 163 | 21.0 |
| 70 to 79 | 68 | 19.3 | 97 | 22.9 | 165 | 21.3 |
| 80+ | 101 | 28.7 | 103 | 24.3 | 204 | 26.3 |
| Area of death | | | | | | |
| Urban | 334 | 43.0 | 397 | 51.2 | 731 | 94.2 |
| Rural | 18 | 2.3 | 27 | 3.5 | 27 | 5.8 |
| Place of death | | | | | | |
| Hospital/clinic | 268 | 34.5 | 341 | 43.9 | 609 | 78.5 |
| Health center/post | 1 | 0.1 | 1 | 0.1 | 2 | 0.3 |
| Home | 79 | 10.2 | 75 | 9.7 | 154 | 19.8 |
| Workplace | 0 | 0.0 | 3 | 0.4 | 3 | 0.4 |
| Another place | 4 | 0.5 | 4 | 0.5 | 8 | 1.0 |
| Determination of cause of death | | | | | | |
| Necropsy | 0 | 0.0 | 3 | 0.4 | 3 | 0.4 |
| Clinical record | 337 | 43.4 | 392 | 50.5 | 729 | 93.9 |
| Interview to relatives or witnesses | 32 | 4.1 | 40 | 5.2 | 72 | 9.3 |
| Clinical history/Lab tests/ interview to relatives or witnesses/Necropsy | 6 | 0.8 | 1 | 0.1 | 7 | 0.9 |

SD: standard deviation

Noteworthy is the stratification of the underlying CoD between original and gold standard death certificates in table 2. In 18 instances where the gold standard certificate identified "COVID-19" as the underlying CoD, the original certificate asserted "diseases of the respiratory system” (Chapter X). Conversely, in six cases where gold standard certificate pinpointed “respiratory system diseases” (Chapter X), the original certificate indicated “COVID-19” (Chapter XXII). Notable discrepancies were also discerned in eight cases where the gold standard certificate specified “circulatory system diseases” (Chapter IX) as the underlying CoD, while the original certificate pointed “endocrine diseases” (Chapter IV). Additionally, ten cases whith "neoplasms" (Chapter II) as the gold standard certificate’s CoD, were classified as "circulatory system diseases" (Chapter IX) in the original certificate.

**Table 2. Cross tabulation of underlying CoD (ICD-10) in original and gold standard death certificates. Colombia, 2021**

| | | Original death certificated | | | | | | | | | | | | | | | | | |
|---|---|---|---|---|---|---|---|---|---|---|---|---|---|---|---|---|---|---|---|
| | ICD-10 chapter | I | II | III | IV | V | VI | IX | X | XI | XII | XIII | XIV | XV | XVI | XVII | XVIII | XX | XXII | Total |
| Standard death certificate | I | 15 | 1 | | | | | 2 | | 2 | | | | | | | 2 | | 1 | 23 |
| | II | 1 | 109 | | | | 1 | 10 | | 3 | 1 | | 1 | | | | | 1 | 1 | 128 |
| | III | | | 5 | | | | | | 2 | | | | | | | | | | 7 |
| | IV | 1 | | | 17 | | | 5 | | | | | | | | | | | 2 | 25 |
| | V | | | | | 2 | | | | | | | 1 | | | | | | | 3 |
| | VI | | | | | 1 | 13 | 3 | | | | | | | | | | | 2 | 19 |
| | IX | | 4 | 1 | 8 | | 3 | 130 | 6 | 5 | | 1 | 4 | 1 | | 1 | 3 | | 5 | 172 |
| | X | 1 | 3 | 1 | 3 | | 1 | 8 | 26 | 1 | | | | 1 | | 1 | | | 6 | 52 |
| | XI | 1 | 1 | | 2 | | | 5 | 2 | 24 | | | | | | | | | | 35 |
| | XII | | | | | | | | 1 | | 1 | | | | | | 1 | | | 3 |
| | XIII | | | | | | | 1 | | | | 3 | | | | | | | 1 | 5 |
| | XIV | 1 | 2 | | 5 | | | 3 | 1 | 1 | | 1 | 5 | | | | 1 | | | 20 |
| | XV | | | | | | | | | | | | | 0 | | | | | | 0 |
| | XVI | 2 | | | | 1 | | | | | | | | | 6 | 1 | | | | 10 |
| | XVII | | | | | | | 2 | | | | | | | 1 | 6 | | | | 9 |

| | | | | | | | | | | | | | | | | | | | |
|---|---|---|---|---|---|---|---|---|---|---|---|---|---|---|---|---|---|---|---|
| **XVIII** | | | | 1 | | | 3 | 1 | | | | | | | | 0 | | | 5 |
| **XX** | | 1 | | | | 1 | 6 | 2 | 1 | | | | | | | | 1 | 1 | 13 |
| **XXII** | 2 | 2 | | 2 | | | 7 | 18 | 1 | | 1 | 2 | | | | | | 212 | 247 |
| **Total** | **24** | **123** | **7** | **38** | **4** | **19** | **185** | **57** | **40** | **2** | **6** | **13** | **2** | **7** | **9** | **7** | **2** | **231** | **776** |

Chapter I: Infectious and parasitic diseases; Chapter II: Neoplasms; Chapter III: Diseases of the blood; Chapter IV: Endocrine diseases; Chapter V: Mental disorders; Chapter VI: Diseases of the nervous system; Chapter IX: Diseases of the circulatory system; Chapter X: Diseases of the respiratory system; Chapter XI: Diseases of the digestive system; Chapter XII: Skin diseases; Chapter XIII: Diseases of the osteomuscular tissue and connective tissue; Chapter XIV: Diseases of the genitourinary system; Chapter XV: Pregnancy, childbirth and puerperium; Chapter XVI: Certain conditions originating in the perinatal period; Chapter XVII: Congenital malformations, deformities and congenital anomalies; Chapter XVIII: Signs, symptoms and poorly defined conditions; Chapter XX: External causes; Chapter XXII: Codes for special situations (COVID-19).

---

Most CoDs reporting “neoplasms” (Chapter II) or “codes for special situations (COVID-19)” (Chapter XXII) exhibited high consistency between the two sources (Table 3). However, conditions such as “diseases of the genitourinary system” (Chapter XIV) showed elevated rates of false positives (Kappa 0.29; 95% CI: 0.08 - 0.50), while "diseases of the circulatory system"(Chapter IX) (Kappa 0.65; 95% CI: 0.58 - 0.71), “codes for special situations (COVID-19)” (Chapter XXII) (Kappa 0.84; 95% CI: 0.79 - 0.88), and “diseases of the respiratory system”(Chapter X) (Kappa 0.44; 95% CI: 0.32 - 0.56) displayed substantial false negatives (Table 3). Greatest variations were for "codes for special situations (COVID-19)" (chapter XXII) (6.9%), "diseases of the circulatory system" (Chapter IX) (-7.0%) and "endocrine diseases, nutritional and metabolic” (Chapter IV) (-34.2%).

**Table 3. Underlying CoD (ICD-10) agreement metrics between original and gold standard death certificates. Colombia, 2021**

| ICD-10 chapter | Death numbers | | Match between certificates | False positive % | False negative % | Kappa | SE | Kappa CI 95% | | Change in Cause-Specific Mortality Fraction (%) |
|---|---|---|---|---|---|---|---|---|---|---|
| | Original (O) | Standard (S) | | | | | | LL | UL | |
| I | 24 | 23 | 15 | 37.5 | 1.1 | 0.63 | 0.084 | 0.46 | 0.79 | -4.2 |
| II | 123 | 128 | 109 | 11.4 | 2.9 | 0.84 | 0.027 | 0.79 | 0.90 | 4.1 |
| III | 7 | 7 | 5 | 28.6 | 0.3 | 0.71 | 0.138 | 0.44 | 0.98 | 0.0 |
| IV | 38 | 25 | 17 | 55.3 | 1.1 | 0.52 | 0.078 | 0.37 | 0.67 | -34.2 |
| V | 4 | 3 | 2 | 50.0 | 0.1 | 0.57 | | 0.13 | 1.00 | -25.0 |
| VI | 19 | 19 | 13 | 31.6 | 0.8 | 0.68 | 0.088 | 0.50 | 0.85 | 0.0 |
| IX | 185 | 172 | 130 | 29.7 | 7.1 | 0.65 | 0.033 | 0.58 | 0.71 | -7.0 |
| X | 57 | 52 | 26 | 54.4 | 3.6 | 0.44 | 0.062 | 0.32 | 0.56 | -8.8 |
| XI | 40 | 35 | 24 | 40.0 | 1.5 | 0.62 | 0.067 | 0.49 | 0.75 | -12.5 |
| XII | 2 | 3 | 1 | 50.0 | 0.3 | 0.40 | 0.278 | 0.00 | 0.94 | 50.0 |
| XIII | 6 | 5 | 3 | 50.0 | 0.3 | 0.54 | 0.182 | 0.19 | 0.90 | -16.7 |
| XIV | 13 | 20 | 5 | 61.5 | 2.0 | 0.29 | 0.105 | 0.08 | 0.50 | 53.8 |
| XV | 2 | 0 | 0 | 100.0 | 0.0 | 0.00 | 0.000 | 0.00 | 0.00 | -100.0 |
| XVI | 7 | 10 | 6 | 14.3 | 0.5 | 0.70 | 0.127 | 0.45 | 0.95 | 42.9 |
| XVII | 9 | 9 | 6 | 33.3 | 0.4 | 0.66 | 0.129 | 0.41 | 0.92 | 0.0 |
| XVIII | 7 | 5 | 0 | 100.0 | 0.7 | -0.01 | 0.002 | -0.01 | 0.00 | -28.6 |
| XX | 2 | 13 | 1 | 50.0 | 1.6 | 0.13 | 0.119 | 0.00 | 0.36 | 550.0 |
| XXII | 231 | 247 | 212 | 8.2 | 6.4 | 0.84 | 0.021 | 0.79 | 0.88 | 6.9 |

SE: standard error LL: lower limit UL: upper limit

---

The proportion of agreement for most CoDs remained consistent between genders, in men was 73% (311 deaths) and in women was 75% (264 deaths). Notably, "neoplasms" (Chapter II) (Kappa 0.82; 95% CI: 0.73 - 0.89 and Kappa 0.87; 95% CI: 0.80 - 0.94, respectively) and "codes for special situations (COVID-19)" (Chapter XXII) (Kappa 0.83; 95% CI: 0.77 - 0.88 and Kappa 0.84; 95% CI: 0.77 - 0.91, respectively), demonstrated almost perfect concordance in both genders. However, "circulatory system diseases" (Chapter IX) exhibited higher percentages of false negatives (Kappa 0.60; 95% CI: 0.49 - 0.69 and Kappa 0.68; 95% CI: 0.60 - 0.76, respectively) (Table 4).

In terms of CSMF attributed to specific CoD, notable variations were identified in both men and women, with the most significant discrepancies observed in “codes for special situations (COVID-19)” (Chapter XXII). Moreover, noteworthy shifts were noted among men, specifically in "endocrine diseases" (Chapter IV) with an increase of +2.2%, and "respiratory system diseases" (Chapter X) also exhibiting a rise of +2.2%. Conversely, women demonstrated substantial variations, particularly in “circulatory system diseases” (Chapter IX) where there was a marked increase of +5.0%.

**Table 4. Underlying CoD (ICD-10) agreement metrics between original and gold standard death certificates by sex. Colombia, 2021.**

| | Men | | | | | | | | | Women | | | | | | | | |
|---|---|---|---|---|---|---|---|---|---|---|---|---|---|---|---|---|---|---|
| ICD-10 chapter | Death numbers | | Match between certificates | False positive % | False negative % | Kappa | Kappa CI95% | | Change in Cause-Specific Mortality Fraction (%) | Death numbers | | Match between certificates | False positive % | False negative % | Kappa | Kappa CI95% | | Change in Cause-Specific Mortality Fraction (%) |
| | Original (O) | Standard (S) | | | | | LL | LS | | Original (O) | Standard (S) | | | | | LL | LS | |
| **I** | 13 | 10 | 8 | 1.21 | 20.00 | 0.69 | 0.47 | 0.91 | -23.1 | 11 | 13 | 7 | 1.18 | 46.15 | 0.57 | 0.33 | 0.81 | 18.2 |
| **II** | 64 | 64 | 54 | 2.78 | 15.63 | 0.82 | 0.73 | 0.89 | 0.0 | 59 | 64 | 55 | 1.39 | 14.06 | 0.87 | 0.80 | 0.94 | 8.5 |
| **III** | 3 | 3 | 2 | 0.24 | 33.33 | 0.66 | 0.23 | 1.00 | 0.0 | 4 | 4 | 3 | 0.29 | 25.00 | 0.75 | 0.41 | 1.00 | 0.00 |
| **IV** | 18 | 8 | 5 | 3.13 | 37.50 | 0.37 | 0.13 | 0.61 | -55.6 | 20 | 17 | 12 | 2.39 | 29.41 | 0.63 | 0.44 | 0.82 | -15.0 |
| **V** | 1 | 0 | 0 | 0.24 | - | 0.00 | 0.00 | 0.00 | -100.0 | 3 | 3 | 2 | 0.29 | 33.33 | 0.66 | 0.23 | 1.00 | 0.0 |
| **VI** | 10 | 10 | 6 | 0.97 | 40.00 | 0.59 | 0.33 | 0.85 | 0.0 | 9 | 9 | 7 | 0.58 | 22.22 | 0.77 | 0.56 | 0.99 | 0.0 |
| **IX** | 72 | 78 | 50 | 6.36 | 35.90 | 0.60 | 0.49 | 0.69 | 8.3 | 113 | 94 | 80 | 12.79 | 14.89 | 0.68 | 0.60 | 0.76 | -16.8 |
| **X** | 37 | 28 | 16 | 5.30 | 42.86 | 0.45 | 0.29 | 0.61 | -24.3 | 20 | 20 | 10 | 3.01 | 50.00 | 0.47 | 0.27 | 0.68 | 0.0 |
| **XI** | 20 | 18 | 13 | 1.72 | 27.78 | 0.67 | 0.49 | 0.84 | -10.0 | 20 | 17 | 11 | 2.69 | 35.29 | 0.57 | 0.37 | 0.77 | -15.0 |
| **XII** | 1 | 1 | 0 | 0.24 | 100.00 | 0.00 | -0.01 | 0.00 | 0.0 | 1 | 2 | 1 | 0.00 | 50.00 | 0.67 | 0.05 | 1.00 | 100.0 |
| **XIII** | 2 | 1 | 1 | 0.24 | 0.00 | 0.67 | 0.05 | 1.00 | -50.0 | 4 | 4 | 2 | 0.57 | 50.00 | 0.49 | 0.07 | 0.92 | 0.0 |
| **XIV** | 8 | 14 | 3 | 1.22 | 78.57 | 0.25 | 0.01 | 0.50 | 75.0 | 5 | 6 | 2 | 0.87 | 66.67 | 0.35 | -0.01 | 0.72 | 20.0 |
| **XV** | 0 | 0 | 0 | 0.00 | - | - | - | - | - | 2 | 0 | 0 | 0.57 | - | 0.00 | - | - | -100.0 |
| **XVI** | 5 | 7 | 4 | 0.24 | 42.86 | 0.66 | 0.35 | 0.97 | 40.0 | 2 | 3 | 2 | 0.00 | 33.33 | 0.80 | 0.41 | 1.00 | 50.0 |
| **XVII** | 6 | 6 | 4 | 0.48 | 33.33 | 0.66 | 0.35 | 0.97 | 0.0 | 3 | 3 | 2 | 0.29 | 33.33 | 0.66 | 0.23 | 1.00 | 0.0 |

| | | | | | | | | | | | | | | | | | | |
|---|---|---|---|---|---|---|---|---|---|---|---|---|---|---|---|---|---|---|
| **XVIII** | 5 | 3 | 0 | 1.19 | 100.00 | -0.01 | -0.01 | 0.00 | -40.0 | 2 | 2 | 0 | 0.57 | 100.00 | -0.01 | -0.01 | 0.00 | 0.0 |
| **XX** | 2 | 8 | 1 | 0.24 | 87.50 | 0.19 | -0.14 | 0.52 | 300.0 | 0 | 5 | 0 | 0.00 | 100.00 | 0.00 | 0.00 | 0.00 | - |
| **XXII** | 157 | 165 | 144 | 5.02 | 12.73 | 0.83 | 0.77 | 0.88 | 5.1 | 74 | 82 | 68 | 2.22 | 17.07 | 0.84 | 0.77 | 0.91 | 10.8 |

220

The Bland-Altman diagram, illustration the absolute difference in underlying CoD between the original and gold standard death certificates (Fig 1), indicates a mean difference was 0 (with limits of agreement: 3.5 to -3.5). The variable plotted in the Bland-Altman diagram was the comparison of underlying CoD grouped by ICD-10 chapter from both the original and gold standard death certificates.

**Fig 1. Bland-Altman plot. Difference between original and gold standard death certificates for underlying CoD.**

# Discussion

This study explores the complexities of the death certification process in Colombia, offering valuable insights into the accuracy of Cause of Death (CoD) reporting. By examining a substantial sample size of 776 randomly selected death certificates from 2021, the second year of the COVID-19 pandemic. Our research employed a meticulous retrospective review, drawing upon information documented in medical records. The collaboration with health professionals adept in death certification preparation and CoD assignment added depth to our analysis. The overall agreement between both the original and gold standard death certificates for the underlying CoD information, categorized according to the ICD-10 chapter, stood at a commendable 74%. Further stratification by gender revealed similar accordance in women (75%) compared to men (73%). These findings underscore the nuance landscape of CoD estimation in Colombia, where both overestimation and underestimation contribute to the complexity of mortality statistics.

Comparing our results globally, the accuracy of the underlying CoD in our study surpassed figures reported in Thailand (37%) (21) and Bangladesh (54%) (22), signifying a comparatively higher precision in Colombia. However, it fell short of the 81% observed in Valparaíso and Metropolitan region in Chile (23). Disparities in age demographics and the

unique challenges posed by the ongoing pandemic caution against direct comparisons, emphasizing the need for context-specific assessments.

Our study highlighted notable overestimations and underestimations in specific CoD categories. High concordance was evident for "neoplasms" (Chapter II) and "codes for special situations (COVID-19)" (Chapter XXII), which collectively constituted 33% of deaths in Colombia during 2021. Overestimation (a high percentages of false negatives and negative values in the CSMF) was identified for "circulatory system diseases" (Chapter IX); "pregnancy, childbirth and puerperium" (Chapter XV); "signs, symptoms, and poorly defined conditions" (Chapter XVIII) and "diseases of the respiratory system" (Chapter X), while underestimation (a high percentages of CSMF) in "diseases of the genitourinary system" (Chapter XIV) among CoD.

The high level of concordance for deaths related to neoplasms, evidenced in the present research, coincides with an earlier study, where 93% of mortality from cancer in Colombia was well certified (16). A Mexican analysis showed that some types of cancer present a better concordance (24). The high concordance with COVID-19 diagnoses could be due to the standardized form that this new diagnosis was added to the list, as it includes information on the condition that started the causal chain of death (25).

The observed discrepancies in CoD assignment can be contextualized within the broader challenges of death certification in Colombia. A substantial proportion of death certificates (79%) were prepared in health services by doctors, in some cases by health professionals with limited experience in this specific task or with a greater workload, which influences in not carefully reviewing the medical records to formulate the CoD sequence (26). Regarding deaths at home, some lack complete data in the medical records, experiencing greater difficulty in determining CoDs, and relatives may not provide enough information. In rural

areas, certification is carried out by other untrained health professionals, introducing possible errors in the certification process (27).

Certification disparities exist between the certification of violent and non-violent deaths. Non-violent deaths are certified by the treating physician or other health professionals authorized to certify them, and violent deaths are certified by the forensic doctor (28). In terms of legal connotations of death certificates, it is true that the certifier must recognize the potential effects that the logical elaboration of the CoD will have. Using certain CoDs may provide information that could be used to challenge, reduce, or deny life insurance benefits. Additionally, the death certificate is rarely used as the sole source of information for legal or insurance purposes. Nevertheless, to reduce or avoid these situations, it is crucial to obtain correct and accurate information at all times (29).

This analysis has limitations. First, the determination of CoD was restricted to the information available in medical records, which may also be imperfect. Second, in deaths where the clinical documentation was incomplete or in the event of a death that occurred at home, relatives or acquaintances of the deceased were interviewed, presenting possible recall bias. Third, potential observer bias in the health professionals who analyzed the medical records, due to the subjectivity. However, the risk of bias is low because the health professionals who analyzed the medical records were trained and were under permanent supervision in the information collection process.

This study had methodological strengths such as the use of a gold standard death certificate, completed by trained health professionals. Additionally, it was based on solid criteria for the selection of cases in multiple jurisdictions at the national level, relying on complete medical records or complemented with information given by relatives, representing a more precise analysis of the death certification process in Colombia. The differences between some of the CoDs identified between the original and gold standard death certificates are associated

with the heterogeneity of the territories, where the death certification process varies depending on factors such as the lack of training of health professionals, incomplete medical records, difficulties in determining the CoDs, or internet access (15,24,28).

In conclusion, our study sheds light on the commendable quality of underlying CoD information in Colombian death certificates, reflecting a 74% concordance with the gold standard death certificate. To enhance the accuracy of the death certification process, we recommend robust educational and training initiatives for health professionals, the development of comprehensive guidelines for death certificate preparation, and ongoing monitoring in health services to identify and address information quality issue. These measures are imperative for refining mortality statistics and ensuring the reliability of CoD data in the Colombian context.

# Acknowledgments

Special thanks to Dr. Richard Garfield of the Centers for Disease Control and Prevention for his valuable technical support during the research development.

# References

1. AbouZahr C, Bratschi MW, Cercone E, Mangharam A, Savigny DD, Dincu I, et al. The COVID-19 Pandemic: Effects on Civil Registration of Births and Deaths and on Availability and Utility of Vital Events Data. Am J Public Health. 2021;111(6):1123-31. doi: 10.2105/AJPH.2021.306203.

2. AbouZahr C, De Savigny D, Mikkelsen L, Setel PW, Lozano R, Nichols E, et al. Civil registration and vital statistics: progress in the data revolution for counting and accountability. The Lancet. 2015;386(10001):1373-85. doi:10.1016/S0140-6736(15)60173-8.

3. AbouZahr C, Mathenge,Gloria, Brøndsted Sejersen T, Macfarlane SB. Civil Registration and Vital Statistics: A Unique Source of Data for Policy. The Palgrave Handbook of Global Health Data Methods for Policy and Practice. 2019;125-44.

4. Ling SM, Warner M, Anderson RN. Opportunities to Enhance the Utility of Cause of Death Information From Death Certificates. Am J Public Health. 2022;112(S1):S42-4. doi:10.2105/AJPH.2022.306722.

5. Merlin JS, Wachterman MW, Becker WC, Bohnert ASB. Serious Illness and Limitations of Death Certificate–Based Data: Urgent Lessons From the Opioid Epidemic. Am J Public Health. 2022;112(S1):S36-8. doi:10.2105/AJPH.2021.306409.

6. Centre of Excellence for CRVS Systems. Why CRVS systems are essential to achieving the Sustainable Development Goals. [Internet]. 2022. Available in: https://crvssystems.ca/index.php/

7. Filippatos G, Andriopoulos P, Panoutsopoulos G, Zyga S, Souliotis K, Gennimata V, et al. The quality of death certification practice in Greece. Hippokratia. 2016;20(1):19-25.

8. Hart JD, Sorchik R, Bo KS, Chowdhury HR, Gamage S, Joshi R, et al. Improving medical certification of cause of death: effective strategies and approaches based on experiences from the Data for Health Initiative. BMC Med. 2020;18(1):74. doi: 10.1186/s12916-020-01519-8.

9. França E, De Abreu DX, Rao C, Lopez AD. Evaluation of cause-of-death statistics for Brazil, 2002–2004. International Journal of Epidemiology. 2008;37(4):891-901. doi:10.1093/ije/dyn121.

10. Mikkelsen L, Phillips DE, AbouZahr C, Setel PW, De Savigny D, Lozano R, et al. A global assessment of civil registration and vital statistics systems: monitoring data quality and progress. The Lancet. 2015;386(10001):1395-406. doi: 10.1016/S0140-6736(15)60171-4.

11. Iburg KM, Mikkelsen L, Adair T, Lopez AD. Are cause of death data fit for purpose? evidence from 20 countries at different levels of socio-economic development. PLoS ONE. 2020;1:15. doi: 10.1371/journal.pone.0237539. doi: 10.1371/journal.pone.0237539.

12. OPS. Portal de indicadores básicos [Internet]. 2022. Available in: https://opendata.paho.org/es/indicadores-basicos/visualizaciones

13. Chaparro-Narváez PE, Jiménez-Serna MM, De La Hoz-Restrepo F. Identificación de los conocimientos de calidad de la información de mortalidad en Bogotá. Revista de salud pública. 2017;19(6):766-71. doi: 10.15446/rsap.V19n6.35377.

14. Cendales R, Pardo C. Quality of death certification in Colombia. Colomb Med. 2018;49(1):121-7. doi: 10.25100/cm.v49i1.3155.

15. Montalvo-Arce CA, Kelly-Forbes YS, Secretaría de Salud San Andrés. Evaluación de la calidad de los certificados de defunción del Hospital Amor de Patria, de San Andrés Islas, Colombia, en 2014. Revista Facultad Nacional de Salud Pública. 2018;36(3):61-70.

16. Cendales R, Pardo C. La calidad de certificación de la mortalidad en Colombia, 2002-2006. Revista de Salud Pública. 2011;13(2):229-38.

17. Rodríguez García J, Vega Landaeta AP, Jaramillo LF, Fernández Ortiz Y, Suarez Morales ZB, Vásquez Candia ME. Calidad en la certificación de mortalidad y natalidad en

un hospital universitario, Colombia año 2014. Revista Gerencia y Políticas de Salud. 2019;18:1-25.

18. Chaparro Narváez PE. Calidad de la información de mortalidad en menores de un año en Bogotá durante 2005 [Internet]. [Bogotá, D.C]: Universidad Nacional de Colombia; 2013. Available in: https://repositorio.unal.edu.co/bitstream/handle/unal/20099/597600.2013.pdf?sequence=1&isAllowed=y

19. Landis JR, Koch GG. The measurement of observer agreement for categorical data. Biometrics.1977;33(1):159-74.

20. Rampatige R, Gamage S, Peiris S, Lopez AD. Assessing the Reliability of Causes of Death Reported by the Vital Registration System in Sri Lanka: Medical Records Review in Colombo. HIM J. 2013;42(3):20-8. doi: 10.1177/183335831304200302.

21. Washirasaksiri C, Raksasagulwong P, Chouriyagune C, Phisalprapa P, Srivanichakorn W. Accuracy and the factors influencing the accuracy of death certificates completed by first-year general practitioners in Thailand. BMC Health Serv Res. 2018;18(1):478. doi: 10.1186/s12913-018-3289-1.

22. Hazard RH, Chowdhury HR, Adair T, Ansar A, Quaiyum Rahman AM, Alam S, et al. The quality of medical death certification of cause of death in hospitals in rural Bangladesh: impact of introducing the International Form of Medical Certificate of Cause of Death. BMC Health Serv Res. 2017;17(1):688. doi: 10.1186/s12913-017-2628-y.

23. Antini C, Rajs D, Muñoz-Quezada MT, Mondaca BAL, Heiss G. Reliability of cause of death coding: an international comparison. Cad Saúde Pública. 2015;31(7):1473-82. doi: 10.1590/0102-311X00099814.

24. Hernández B, Ramírez-Villalobos D, Romero M, Gómez S, Atkinson C, Lozano R. Assessing quality of medical death certification: Concordance between gold standard diagnosis and underlying cause of death in selected Mexican hospitals. Popul Health Metrics. 2011;9(1):38. doi: 10.1186/1478-7954-9-38.

25. Armstrong D. The COVID-19 pandemic and cause of death. Sociology Health & Illness. 2021;43(7):1614-26. doi: 10.1111/1467-9566.13347.

26. Pritt BS, Hardin NJ, Richmond JA, Shapiro SL. Death Certification Errors at an Academic Institution. Archives of Pathology & Laboratory Medicine. 2005;129(11):1476-9. doi: 10.5858/2005-129-1476-DCEAAA.

27.Vargas Lorenzo I. Barreras en el acceso a la atención en salud en modelos de competencia gestionada: un estudio de caso en Colombia. Universidad Autónoma de Barcelona. 2009. Available in: https://www.tdx.cat/handle/10803/4651

28. Juyal D, Kumar A, Pal S, Thaledi S, Jauhari S, Thawani V. Medical certification of cause of death during COVID-19 pandemic - a challenging scenario. J Family Med Prim Care. 2020 Dec 31;9(12):5896-5898. doi: 10.4103/jfmpc.jfmpc_1435_20. PMID: 33681015; PMCID: PMC7928126.

29. National Center for Health Statistics. Cause of Death and the Death Certificate Important Information for Physicians. Department of Health and Human Services. 1997.Available in: https://www.cdc.gov/nchs/nvss/writing-cod-statements/death_certification_problems.htm.

# DATABASE ACCURACY

All relevant data are within the manuscript and its Supporting Information files.

Figure

Click here to access/download;Figure;Fig1.eps.eps

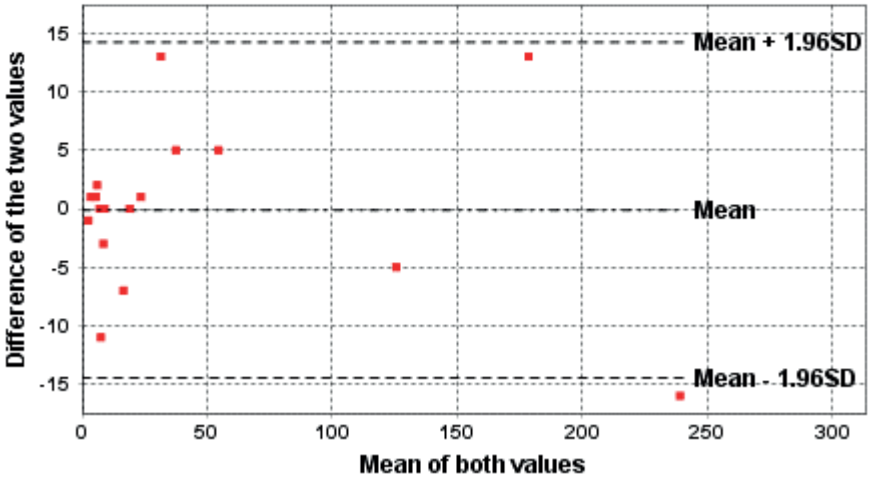

Click here to access/download

**Supporting Information**

Database Accuracy..xlsx

# Accuracy of ~~the I~~Iinformation on the ~~basic~~ U~~u~~nderlying C~~c~~ause of D~~d~~eath: A~~a~~n A~~a~~nalysis in Colombia during the COVID-19 P~~p~~andemic in 2021

Pablo Chaparro-Narváez1¶, Jessika Alexandra Manrique Sanchez1¶, Laura Berrio-Parra1¶ * Diana Carolina Urrego Ricaurte1¶, Luis José Torres-Rojas1¶, ,Nidia Patricia Orjuela Cantor1&, Carlos Castañeda-Orjuela1¶,

**Affiliations**

[1] National Health Observatory, National Institute of Health, Bogotá, Colombia.

*lberrio@ins.gov.co (LB)

## Abstract

~~Introduction~~**Objective**: ~~We~~This study aimed to estimate the accuracy of the underlying Cause of Death (CoD) in the "~~-~~original~~-~~" death certificate (DC), compared with a "~~-~~gold

**Formatted:** Font: 11 pt

**Formatted:** Space After: 0 pt, Line spacing: single, Don't adjust space between Latin and Asian text, Don't adjust space between Asian text and numbers

**Formatted:** Font: 11 pt

**Formatted:** Font: 11 pt

**Formatted:** Font: 11 pt

**Formatted:** Font: 11 pt

**Formatted:** Font: 11 pt

**Formatted:** Font: 11 pt

**Formatted:** Font: Not Bold

**Field Code Changed**

**Formatted:** Heading 2, Justified, Space After: 8 pt, Line spacing: Double

**Formatted:** Font: Bold

**Formatted:** Font: Bold

standard certificate" based on information from clinical charts and relatives, in population deceased in Colombia during 2021.

**Methods:** A~~The study~~ sample size of ~~consisted in~~ 806 deaths ~~in accros~~across ~~the~~ 92 municipalities ~~of~~ in Colombia were~~as~~ estimated from the pool of 326,833 "original" certificate~~DCs~~ ~~from~~ provided by the National Department of Statistics. A~~(DANE in Spanish acronym) were selected using a~~ two-stage stratified random sample with replacement was employed for selection. ~~Researchers tookI~~ information from ~~the deceased's~~ medical records of the deceased and, when necessary, interviews with~~ed~~ relatives or witnesses were used to determine CoDs on the "gold standard certificate". We analyzed and compared the underlying CoD of the "original" and "standard" ~~DC~~death certificates to ~~determine~~ estiamate the level of accuracy ~~between DCs~~. M~~, m~~easures of concordance, patterns of false positives and negatives, and calculate a kappa value were utilized as metrics to evaluate the quality of the ~~DC~~death certificates.

**Results:** Information was obtained from 776, representing ~~(~~96% of the desired sample~~)~~ ~~DCs~~. The concordance between "original" and gold "standard" ~~DCs~~certificates, categorized according to the ICD-10 chapters, was found to be 74%. H~~A h~~igher levels of agreement were observed~~as found~~ for "codes for special COVID-19 situations" (kappa=0. 84) and neoplasms (kappa=0.~~84~~84). Higher levels of agreement were observed for "codes for special COVID-19 situations" (kappa=0.84) and neoplasms (kappa=0.84). Overestimation was identified for "circulatory system diseases" (Chapter IX); "pregnancy, childbirth and puerperium" (Chapter XV); "signs, symptoms, and poorly defined conditions" (Chapter XVIII) and "diseases of the respiratory system" (Chapter X), while underestimation in "diseases of the genitourinary system" (Chapter XIV) among CoD.

**Conclusions:** The level of concordance between the "original" and "gold standard" death certificate~~DCs~~ was deemed ~~good~~adequate. However, ~~It´s convenient to~~ improvements in

**Formatted:** Font: Arial
**Formatted:** Font: Arial
**Formatted:** Font: Arial
**Formatted:** Font: Arial
**Formatted:** Font: Arial
**Formatted:** Font: Arial
**Formatted:** Font: Arial
**Formatted:** Font: Arial
**Formatted:** Font: Arial
**Formatted:** Font: Arial
**Formatted:** Font: Arial
**Formatted:** Font: Arial
**Formatted:** Font: Arial
**Formatted:** Font: Arial
**Formatted:** Font: Arial
**Formatted:** Font: Arial
**Formatted:** Font: Arial
**Formatted:** Font: Arial, Bold
**Formatted:** Font: Arial
**Formatted:** Font: Arial
**Formatted:** Font: Arial
**Formatted:** Font: Arial
**Formatted:** Font: Arial
**Formatted:** Font: Arial
**Formatted:** Font: Arial

the death certification process in Colombia are recommended, ~~strengthening~~ emphasizing the enhancement of training programs ~~o~~for health professionals.

***Keywords:*** *Underlying cause of death; Death certificate; Medical information; Data accuracy; Colombia.*

# Introduction

The global impact of the COVID-19 pandemic ~~highlighted~~ underscored the ~~weaknesses~~ vulnerabilities inherent in ~~of~~ Civil Registration and Vital Statistics (CRVS) systems ~~around the~~ worldwide, ~~and demonstrated~~emphasizing the ~~need~~ imperative to ~~improve~~ enhance information systems (1). Effective~~Good~~ public policy formulation rel~~qu~~i~~r~~es on comprehensive, detailed, continuous, and real-time statistics related to~~n~~ population health and mortality, ~~among other aspects, information that is~~which are integral components provided by the CRVS system (2).

Encompassing vital events such as births, deaths, and others, t~~T~~he CRVS system not only yields ~~accounts for vital events including births, deaths and others., marriages, divorces, and adoptions. The CRVS system provides~~ legal and administrative ~~benefits~~ advantages for individuals and families~~,~~ ~~and~~ but also supplies crucial statistical or administrative information for policymakers. ~~For the former, the registration of deaths is essential for inheritance claims, insurance and benefits for the spouse and survivors. For the latter,~~ C~~The c~~ontinuous registration of deaths, alon~~g~~~~d~~ with their causes, serves as a cor~~a~~nerstone ~~guide~~ for informed policy formulation aimed at~~ies to~~ reducing~~e~~ premature mortality. In ~~weak~~ systems with inherent weaknesses, the response to ~~many deaths can occur before~~ health ~~response to a~~ threats ~~are~~ may be ~~mobilized~~delayed, resulting ~~un~~in a higher incidence of deaths. C~~By c~~onversely,~~trast~~ timely mortality statistics enable ~~mortality~~ surveillance, consider~~tak~~ing ~~into account~~the evolving trend in ~~that considers~~deaths burden

~~trends and the growing burden of deaths from~~ for ~~-~~any cause, ~~and enables~~providing essential up-to-date data ~~essential~~ for informed decision-making (3).

~~CoD~~The information on the Cause of Death (coD) ~~can~~ plays a pivotal role in guiding ~~be used to inform~~ decision-~~-~~making process, ~~in many aspects of public life,~~ influenc~~clud~~ing policy direction, research funding priorities, ~~practice,~~ and evaluating the ~~impact~~ effectiveness of public health interventions~~.~~ (4,5). Seven ~~of the~~ Sustainable Development Goals (SDGs) ~~utilize~~ specifically rely on mortality data sourced from CRVS systems (6). ~~Additionally~~However, the accuracy and timeliness of data ~~collected and~~ recorded on death certificate~~DC~~s can ~~be quite~~exhibit significant variabilit~~le~~y (7–9).

Deficiencies in CoD information ~~the~~ quality can stem from factor such as ~~of CoD information may be due to a lack of~~inadequate training among coders, unfamiliarity with ~~the~~ causes (7,10), time constraints (6), and a ~~or~~ lack of supervision, leading to the frequent use of imprecise "garbage codes" (11). In a~~A~~dditioned~~ed~~ to these factor~~reason~~s the implementation of effective procedures to consolidate the information~~,~~ and the adherence to statistical standards that transform individual codes into comprehensive national mortality statistics play a crucial role (8). No~~E~~tably, errors in CoD reporting are ~~common~~prevalent, ~~varying~~ with rates ~~rabngies~~ranges between 39% and 61% in population-based studies and ~~between~~ 32% ~~and~~ to 45% in hospital studies (7–9).

Formatted: English (United States)

Concerning the ~~Under~~reporting landscape~~of deaths in 2019~~ in the Americas in 2019, underreporting was estimated at 4%, accompanied by~~while~~ the utilization~~se~~ of poorly defined or absent CoD codes ~~was~~ (3%)~~,~~ and the inclusion of garbage codes (~~in~~ 15%). Conversely,~~In~~ Colombia ~~for the same year~~exhibited~~,~~ higher underreporting ~~was higher,~~ at 13%, with poorly defined and ignored CoD accounting for 1%, and garbage codes for 11% (12). Despite these statistics,

~~In~~ Colombia's ~~the few~~ studies on CoD have been limited, focusing~~examined~~ on aspects such as the knowledge ~~that~~ of different CRVS actors (13)~~-~~, the quality of certification ~~of~~ in general and cancer mortality (14–16), ~~quality in~~ the certification quality of live births and non-fetal deaths in hospitals (15,17), and the quality of ~~the~~ information ~~in~~ for children under one year of age (18). In light of this, our

~~The objective of this~~ study aims to fill this research gap by~~was to~~ estimating~~e~~ the accuracy of underlying CoD in the "original" ~~DC~~death certificate, compared with a "gold standard certificate" based on information from clinical charts and relatives, in population deceased in Colombia during 2021.

# Methods

A cross-sectional descriptive study ~~compared~~ was conducted~~arried out.~~ ~~i~~In Colombia during in 2021. A~~, a~~t the ~~time this~~ study's ~~began~~commencement, the National Department on Statistics (DANE, by the Spanish acronym of *Departamento Administrativo Nacional de Estádística*) had compiled ~~a total of~~ 326,833 non-fetal death certificates~~DC~~s, excluding ~~deaths~~those related ~~due~~ to violent causes certified by the Institute of Legal Medicine and Forensic Sciences, as well as~~;~~ ~~and~~ municipalities ~~in Colombia~~ without reported COVID-19 infections or deaths. The selection criteria for th~~e~~is study ~~were:~~included municipalities ~~of Colombia~~ classified as large and intermediate~~, municipalities in~~ where~~ich~~ cases of COVID-19 were identifi~~detect~~ed based~~,~~ ~~according~~ to the ~~degree of~~ complexity of health care services; and the presence ~~municipalities in Colombia with~~of more than 20 deaths

associated of patients with COVID-19 or from other causes associated with COVID-19, in the period from January to December 2021as, determined in medical records and/or death certificateDC.

A two-stage stratified random sample with replacement was designed. TIn the first stage connsidered, conglomerates made up of thecomprising 33 territorial entities (32 departments and the district of Bogotá) were considered. In the second stage, 92 municipalities were selected, and with the population was stratified by age groups (<60 years and 60 and more years). The sample size was estimated sample size wasin 806 deaths distributed inacross the 92 municipalities of Colombia, with a confidence level (CI) of 95%, an expected proportion of 70%, an estimation precision of 5%, and an effect of 2.5 for the two-stage sampling design.

IThe information was taken from the medical records andwas collected and analyzed by a team of health professionals, including (five doctors and a nurse with a postgraduate degree in epidemiology and public health. The professiionalsprofessional´s underwent ) that prioreviously trainingged in the DANE vital statistics course to ensure , for the correctaccurate ementassignmenting of CoDs in the "gold standard certificate". The review of the medical records was carriedtook place out from between 01/06/2022 toand 30/09/2022.

TBased on the identification data of the deceased´s, as presentcontained in the "original death " DcertificateCs, were used to requestthe corresponding medical records were requested from each of the territorial health entities. These health professionals, under strict supervision, went to different territories and extracted information from these medical records to complete the underlying CoD in the "standard death certificate" DC, considered as the gold standard certificate offor this study. To mitigate bias, health professionals never had access to the original death certificate. In cases where the medical records

**Formatted:** Space After: 8 pt

lacked sufficient information to establish the CoD, interview with family members or acquaintances were interviewed conducted.

Formatted: Font color: Accent 1

The group of health professionals were under strict supervision and training included weekly meetings to review special cases and agree on decision criteria. Health professionals never saw the “original” DC to avoid bias.

Afterwards, an expert nosologist coded the CoD assigned by the health personnel, and selectinged the underlying CoD based on the norms established by the International Classification of Diseases, 10th Revision (ICD-10) norm, which is used in Colombia`sthe official mortality statistics of Colombia. There was no For the attribution of the CoD adjudicating. committee;s, instead, the CoDsthese were directly collected from the medical records by the group of health professionals., Therefore, there wasn´t a CoD adjudicating committee. In cases lacked sufficientinformation to establish interview with family members or acquaintances of the deceased were interviewed conducted. The information collected was processed in spreadsheets of Microsoft Excel and analyzed with Stata, version 12.0

Formatted: English (United States)

The gold s“Standard certificate” DC, based on detailed medical records, was considered deemed the most accurate source. A comparison of tThe CoD on f “original” and gold “standard certificated” DCs wasere conductmpared to identify false positives, false negatives, and kappa value, along with their respective 95% confidence levels interval (95% CI). False positive and false negative percentages wewrwwere used to cuantifyquantify% represents the degree of overdiagnosis of a diseaseand under-certification, respectively by the “original” DC as judged by the “standard” DC. False negative %, expresses the degree of under certification of a disease according to the comparison between the “original” and “standard” DC. Regarding the strength of agreement,K kappa values greater than 0.81 were considered as almost perfect, range

0.61 to 0.80 as high,~~;~~ 0.41 to 0.60 as moderate,~~;~~ 0.21 to 0.40 as acceptable, 0.00 to 0.20 as low, and those < 0.00 as poor (19). The change in cause-specific mortality fraction (CSMF) was also calculated (20) as follows:

$$CSMF = \frac{CaSMF^{S} - CaSMF^{\circ}}{CaSMF^{\circ}} \; x\, 100$$

Where $CaSMF$ = cause specific mortality fraction, O = "original" certificate, S = "standard" certificate.

$$CaSMF = \frac{Number\ of\ deaths\ due\ to\ one\ specific\ cause}{Total\ number\ of\ deaths}$$

Additionally, t~~T~~o asses~~he~~ ~~comparison of~~ variations in the underlying CoDs between original ~~CoD of "original"~~ and gold "standard certificate~~" DCs~~ ~~also used~~ the Bland-Altman diagram~~–~~ was employed to visualize the comparison~~that compared the average and the difference between these certificates.~~ The collected information ~~collected~~ was processed in ~~spreadsheets of~~ Microsoft Excel spreadsheets and analyzed ~~with~~using Stata, version 12.0.

The project ~~was~~received approval~~ed~~ ~~by~~from the Committee on Ethics and Research Methodologies of the National Institute of Health (In Spanish *Comité de Ética y Metodologías~~s~~ de la Investigación* - ~~(~~CEMIN~~)~~). Access to ~~the~~ anonymized mortality databases ~~of the~~from DANE wa~~i~~s ~~open~~granted, and t~~. The~~ extraction of information~~specific procedure consisted in extracting,~~ from the medical records by health professionals was conducted ~~of the deceased´s, the information necessary to complete the "standard" DC. These health professionals didn't have~~without accessing ~~to~~ the CoDs ~~of~~from the "original" ~~DC~~ , certificate to ~~aviod~~prevent potential bias.

**Formatted:** Font: 10 pt

**Formatted:** Font: 10 pt

**Formatted:** Font: 10 pt, English (United States)

**Formatted:** Font: 10 pt

**Formatted:** Font: 9 pt

**Formatted:** Font: 10 pt

**Formatted:** Font: (Default) +Body (Calibri), Not Italic, English (United States)

**Formatted:** Font: +Body (Calibri), 10 pt

**Formatted:** Left, Right: 0", Space Before: 0 pt, After: 8 pt

**Formatted:** English (United States)

**Formatted:** Normal

**Formatted:** Font: 10 pt, Italic

**Formatted:** Space After: 8 pt

**Formatted:** Font: Italic

**Formatted:** Font: Italic

Regard to the medical records, CEMIN also authorized the review of medical records indicated that, in its capacity asin the capacity of the thepublic health authority of, the National Institute of Health was authorized to request the review of these documents, and t. These medical recordsdocuments remained securely uinder the custody of the research team and the respective territorial entities. T

Finally, when necessary, telephone interviews, conducted were realized with previousprior verbal consent, were carried out as required. It was explained to the interlocutor whatDuring tehsethese interviews, the anturenature of the project was about, the information sought, and its intended requested and how it would be use were clearly explained to the participantsd. The purely voluntary nature of their participation was also made explicitemphasized, and. tThe data analyzed was rigorously anonymized. This procedure was endorsed by CEMIN, ensuring ethical standards and the protection of participants' confidentiality throughout the research process.

# Results

OIn our sample of the 806776 deaths in the derived sample, sinformation was obtained from 776 (96% of the sample). The sample was stratification was conducteded by department, municipality, and age, ensuring a . In addition, it is representative cross-section of the total number of deaths in Colombia`s total mortality. The Despite a nominal 4% loss in of 4% of the sample,thissample, this minimal reduction did no´t introduce any produce distortion to theion in thestudy's findingresults of this study.

The table 1 shows the characteristics of included deaths in 2021, the mean age of death was 65 years (range:, 0-98 years; median:, 68 years). Most of the deaths occurred in the urban areas areas (94.2%) and hospitals (78.5%). CoD determination primarily relied on medical records (93.9%), with a 1% of the sample drawing from a multifaceted array of

**Formatted:** Indent: Left: 0", Right: 0"

**Formatted:** Font color: Text 1

**Formatted:** Font: 18 pt

sources such as medical records, laboratory tests and interview with relatives or witnesses (Table 1). Overall, the agreement on the underlying CoD, based on ICD-10, was robust at 74% (575 deaths)

**Table 1. Characteristics of analyzed ~~the~~ death~~ceased included in the analysi~~s. Colombia, 2021**

**Formatted:** English (United States)

| | Women (n = 352) | | Men (n = 424) | | Total (n = 776) | |
|---|---|---|---|---|---|---|
| | n | % | n | % | n | % |
| Age | | | | | | |
| Mean age (SD) at death, in years | 65.9 | 21.4 | 64.4 | 20.9 | 65.1 | 21.1 |
| 0 to 9 | 12 | 3.4 | 15 | 3.5 | 27 | 3.5 |
| 10 to 19 | 2 | 0.6 | 6 | 1.4 | 8 | 1.0 |
| 20 to 29 | 10 | 2.8 | 6 | 1.4 | 16 | 2.1 |
| 30 to 39 | 15 | 4.3 | 21 | 5.0 | 36 | 4.6 |
| 40 to 49 | 22 | 6.3 | 31 | 7.3 | 53 | 6.8 |
| 50 to 59 | 42 | 11.9 | 62 | 14.6 | 104 | 13.4 |
| 60 to 69 | 80 | 22.7 | 83 | 19.6 | 163 | 21.0 |
| 70 to 79 | 68 | 19.3 | 97 | 22.9 | 165 | 21.3 |
| 80+ | 101 | 28.7 | 103 | 24.3 | 204 | 26.3 |
| Area of death | | | | | | |
| Urban | 334 | 43.0 | 397 | 51.2 | 731 | 94.2 |
| Rural | 18 | 2.3 | 27 | 3.5 | 27 | 5.8 |
| Place of death | | | | | | |
| Hospital/clinic | 268 | 34.5 | 341 | 43.9 | 609 | 78.5 |
| Health center/post | 1 | 0.1 | 1 | 0.1 | 2 | 0.3 |
| Home | 79 | 10.2 | 75 | 9.7 | 154 | 19.8 |
| Workplace | 0 | 0.0 | 3 | 0.4 | 3 | 0.4 |
| Another place | 4 | 0.5 | 4 | 0.5 | 8 | 1.0 |
| Determination of cause of death | | | | | | |
| Necropsy | 0 | 0.0 | 3 | 0.4 | 3 | 0.4 |

| | | | | | | |
|---|---|---|---|---|---|---|
| Clinical record | 337 | 43.4 | 392 | 50.5 | 729 | 93.9 |
| Interview to relatives or witnesses | 32 | 4.1 | 40 | 5.2 | 72 | 9.3 |
| Clinical history/Lab tests/ interview to relatives or witnesses/Necropsy | 6 | 0.8 | 1 | 0.1 | 7 | 0.9 |

SD: standard deviation

---

Noteworthy is the stratification of~~The tabulation of~~ the underlying CoD ~~selected~~ between~~in~~ ~~“~~original~~”~~ and gold ~~“~~standard~~”~~ death certificate~~DC~~s ~~is presented~~ in table 2. In 18 instanc~~cas~~es ~~in~~ where~~ich~~ the gold ~~“~~standard certificate~~” DC~~ identifi~~determin~~ed "COVID-19" as the underlying CoD, the ~~“~~original certificate~~” DC~~ assert~~establish~~ed "diseases of the respiratory system” (Chapter X). C~~By c~~onversely~~trast~~, in six cases ~~in~~ where~~ich~~ gold standard certificate ~~found~~ pinpointed “respiratory system diseases” (Chapter X) ~~as the underlying CoD,~~ the ~~“~~original~~” DC~~ certificate indicat~~establish~~ed “COVID-19” (Chapter XXII). Notable d~~D~~iscrepancies were also discern~~observ~~ed in eight cases ~~in~~ where~~ich~~ the gold ~~“~~standard certificate~~” DC~~ specified “circulatory system diseases” (Chapter IX) as the underlying CoD, while ~~and~~ the ~~“~~original certificate~~” DC~~ poin~~indica~~ted “endocrine diseases” (Chapter IV). A~~In a~~dditionally, ~~in~~ ten cases ~~in~~ whi~~t~~ch ~~the “standard” DC specified~~ "neoplasms" (Chapter II) as ~~underlying~~the gold standard certificate’s CoD, ~~the “original” DC assigned~~were classified as "circulatory system diseases" (Chapter IX) in the original certificate.

**Table 2. Cross tabulation of ~~the~~ underlying CoD (ICD-10) in~~of “~~ original~~”~~ and** gold **~~“~~standard~~”~~** death **certificate~~DCs according to the ICD-10~~. Colombia, 2021**

| | | Original death certificated | | | | | | | | | | | | | | | | | |
|---|---|---|---|---|---|---|---|---|---|---|---|---|---|---|---|---|---|---|---|
| | **ICD-10 chapter** | **I** | **II** | **III** | **IV** | **V** | **VI** | **IX** | **X** | **XI** | **XII** | **XIII** | **XIV** | **XV** | **XVI** | **XVII** | **XVIII** | **XX** | **XXII** | **Total** |
| **Standard** | **I** | 15 | 1 | | | | | 2 | | 2 | | | | | | | 2 | | 1 | 23 |
| | **II** | 1 | 109 | | | | 1 | 10 | | 3 | 1 | | 1 | | | | | 1 | 1 | 128 |
| | **III** | | | 5 | | | | | | 2 | | | | | | | | | | 7 |

| | | | | | | | | | | | | | | | | | | | |
|---|---|---|---|---|---|---|---|---|---|---|---|---|---|---|---|---|---|---|
| **IV** | 1 | | | 17 | | | 5 | | | | | | | | | | | 2 | 25 |
| **V** | | | | | 2 | | | | | | | 1 | | | | | | | 3 |
| **VI** | | | | | 1 | 13 | 3 | | | | | | | | | | | 2 | 19 |
| **IX** | | 4 | 1 | 8 | | 3 | 130 | 6 | 5 | | 1 | 4 | 1 | | 1 | 3 | | 5 | 172 |
| **X** | 1 | 3 | 1 | 3 | | 1 | 8 | 26 | 1 | | | | 1 | | 1 | | | 6 | 52 |
| **XI** | 1 | 1 | | 2 | | | 5 | 2 | 24 | | | | | | | | | | 35 |
| **XII** | | | | | | | | 1 | | 1 | | | | | | 1 | | | 3 |
| **XIII** | | | | | | | 1 | | | | 3 | | | | | | | 1 | 5 |
| **XIV** | 1 | 2 | | 5 | | | 3 | 1 | 1 | | 1 | 5 | | | | 1 | | | 20 |
| **XV** | | | | | | | | | | | | | 0 | | | | | | 0 |
| **XVI** | 2 | | | | 1 | | | | | | | | | 6 | 1 | | | | 10 |
| **XVII** | | | | | | | 2 | | | | | | | 1 | 6 | | | | 9 |
| **XVIII** | | | | 1 | | | 3 | 1 | | | | | | | | 0 | | | 5 |
| **XX** | | 1 | | | | 1 | 6 | 2 | 1 | | | | | | | | 1 | 1 | 13 |
| **XXII** | 2 | 2 | | 2 | | | 7 | 18 | 1 | | 1 | 2 | | | | | | 212 | 247 |
| **Total** | **24** | **123** | **7** | **38** | **4** | **19** | **185** | **57** | **40** | **2** | **6** | **13** | **2** | **7** | **9** | **7** | **2** | **231** | **776** |

Chapter I: Infectious and parasitic diseases; Chapter II: Neoplasms; Chapter III: Diseases of the blood; Chapter IV: Endocrine diseases; Chapter V: Mental disorders; Chapter VI: Diseases of the nervous system; Chapter IX: Diseases of the circulatory system; Chapter X: Diseases of the respiratory system; Chapter XI: Diseases of the digestive system; Chapter XII: Skin diseases; Chapter XIII: Diseases of the osteomuscular tissue and connective tissue; Chapter XIV: Diseases of the genitourinary system; Chapter XV: Pregnancy, childbirth and puerperium; Chapter XVI: Certain conditions originating in the perinatal period; Chapter XVII: Congenital malformations, deformities and congenital anomalies; Chapter XVIII: Signs, symptoms and poorly defined conditions; Chapter XX: External causes; Chapter XXII: Codes for special situations (COVID-19).

---

M~~Alm~~ost~~ all~~ CoDs reporting "neoplasms" (Chapter II) or "codes for special situations (COVID-19)" (Chapter XXII) ~~were ~~exhibited high consistency~~t~~ ~~across ~~between the two sources (Table 3). However, c~~C~~onditions such as "diseases of the genitourinary system" (Chapter XIV) showe~~ha~~d elevated~~high~~ rates of false positives (Kappa 0.29; 95% CI: 0.08 - 0.50), while~~.~~ "d~~D~~iseases of the circulatory system"(Chapter IX) (Kappa 0.65; 95% CI: 0.58 - 0.71),~~;~~ "codes for special situations (COVID-19)" (Chapter XXII) (Kappa 0.84; 95% CI: 0.79

- 0.88), and “diseases of the respiratory system” (Chapter X) (Kappa 0.44; 95% CI: 0.32 - 0.56) displaye, had high substantial percentages of false negatives (Table 3).

Greatest variations were for "codes for special situations (COVID-19)" (chapter XXII) (6.9%), "diseases of the circulatory system" (Chapter IX) (-7.0%) and "endocrine diseases, nutritional and metabolic” (Chapter IV) (-34.2%). Greatest variations were for "codes for special situations (COVID-19)" (chapter XXII) (6.9%), "diseases of the circulatory system" (Chapter IX) (-7.0%) and "endocrine diseases, nutritional and metabolic” (Chapter IV) (-34.2%).

**Table 3. Underlying CoD (ICD-10) agreement metrics between “original” and gold the “standard” death certificateDCs according to the ICD-10. Colombia, 2021**

| ICD-10 chapter | Death numbers | | Match between certificates | False positive % | False negative % | Kappa | SE | Kappa CI 95% | | Change in Cause-Specific Mortality Fraction (%) |
|---|---|---|---|---|---|---|---|---|---|---|
| | Original (O) | Standard (S) | | | | | | LL | UL | |
| I | 24 | 23 | 15 | 37.5 | 1.1 | 0.63 | 0.084 | 0.46 | 0.79 | -4.2 |
| II | 123 | 128 | 109 | 11.4 | 2.9 | 0.84 | 0.027 | 0.79 | 0.90 | 4.1 |
| III | 7 | 7 | 5 | 28.6 | 0.3 | 0.71 | 0.138 | 0.44 | 0.98 | 0.0 |
| IV | 38 | 25 | 17 | 55.3 | 1.1 | 0.52 | 0.078 | 0.37 | 0.67 | -34.2 |
| V | 4 | 3 | 2 | 50.0 | 0.1 | 0.57 | | 0.13 | 1.00 | -25.0 |
| VI | 19 | 19 | 13 | 31.6 | 0.8 | 0.68 | 0.088 | 0.50 | 0.85 | 0.0 |
| IX | 185 | 172 | 130 | 29.7 | 7.1 | 0.65 | 0.033 | 0.58 | 0.71 | -7.0 |
| X | 57 | 52 | 26 | 54.4 | 3.6 | 0.44 | 0.062 | 0.32 | 0.56 | -8.8 |
| XI | 40 | 35 | 24 | 40.0 | 1.5 | 0.62 | 0.067 | 0.49 | 0.75 | -12.5 |
| XII | 2 | 3 | 1 | 50.0 | 0.3 | 0.40 | 0.278 | 0.00 | 0.94 | 50.0 |
| XIII | 6 | 5 | 3 | 50.0 | 0.3 | 0.54 | 0.182 | 0.19 | 0.90 | -16.7 |
| XIV | 13 | 20 | 5 | 61.5 | 2.0 | 0.29 | 0.105 | 0.08 | 0.50 | 53.8 |
| XV | 2 | 0 | 0 | 100.0 | 0.0 | 0.00 | 0.000 | 0.00 | 0.00 | -100.0 |
| XVI | 7 | 10 | 6 | 14.3 | 0.5 | 0.70 | 0.127 | 0.45 | 0.95 | 42.9 |
| XVII | 9 | 9 | 6 | 33.3 | 0.4 | 0.66 | 0.129 | 0.41 | 0.92 | 0.0 |
| XVIII | 7 | 5 | 0 | 100.0 | 0.7 | -0.01 | 0.002 | -0.01 | 0.00 | -28.6 |

| | | | | | | | | | | |
|---|---|---|---|---|---|---|---|---|---|---|
| XX | 2 | 13 | 1 | 50.0 | 1.6 | 0.13 | 0.119 | 0.00 | 0.36 | 550.0 |
| XXII | 231 | 247 | 212 | 8.2 | 6.4 | 0.84 | 0.021 | 0.79 | 0.88 | 6.9 |

SE: standard error LL: lower limit UL: upper limit

---

The proportion of agreement for most CoDs remained consistent between genders, in men was 73% (311 deaths) and in women was 75% (264 deaths). Notably, "neoplasms" (Chapter II) (Kappa 0.82; 95% CI: 0.73 - 0.89 and Kappa 0.87; 95% CI: 0.80 - 0.94, respectively) and "codes for special situations (COVID-19)" (Chapter XXII) (Kappa 0.83; 95% CI: 0.77 - 0.88 and Kappa 0.84; 95% CI: 0.77 - 0.91, respectively), demonstrated almost perfect concordance in both genders. However, "circulatory system diseases" (Chapter IX) exhibited higher percentages of false negatives (Kappa 0.60; 95% CI: 0.49 - 0.69 and Kappa 0.68; 95% CI: 0.60 - 0.76, respectively) (Table 4).

~~Regarding~~ in terms of~~the~~ CSMF attributed ~~due~~ to specific CoD, ~~the greatest~~notable variations were ~~also~~ identifi~~observ~~ed in both men and women, with the most significant discrepancies observed in “codes for special situations (COVID-19)” (Chapter XXII). ~~In addition~~Moreover, noteworthy shifts were noted ~~other important variations observed in~~among men, specifically ~~were~~ in "endocrine diseases" (Chapter IV) with an increase of ~~(~~+2.2%,~~)~~ and "respiratory system diseases" (Chapter X) ~~(~~also exhibiting a rise of +2.2%. Conversely,~~); while in~~ women ~~were~~ demonstrated substancial variations, particularly in “circulatory system diseases” (Chapter IX) where there was a marked increase of ~~(~~+5.0%~~)~~.

**Table 4. Underlying CoD (ICD-10) agreement metrics between "original" and "gold standard" death certificate~~DC~~s ~~according to the ICD-10~~ by sex. Colombia, 2021.**

| ICD-10 chapter | Men | | | | | | | | | Women | | | | | | | | |
|---|---|---|---|---|---|---|---|---|---|---|---|---|---|---|---|---|---|---|
| | Death numbers | | Match between certificates | False positive % | False negative % | Kappa | Kappa CI95% | | Change in Cause-Specific Mortality Fraction | Death numbers | | Match between certificates | False positive % | False negative % | Kappa | Kappa CI95% | | Change in Cause-Specific Mortality Fraction |
| | Original (O) | Standard (S) | | | | | LL | LS | | Original (O) | Standard (S) | | | | | LL | LS | |

| | | | | | | | | | | | | | | | | | | |
|---|---|---|---|---|---|---|---|---|---|---|---|---|---|---|---|---|---|---|
| | | | | | | | | | (%) | | | | | | | | | (%) |
| I | 13 | 10 | 8 | 1.21 | 20.00 | 0.69 | 0.47 | 0.91 | -23.1 | 11 | 13 | 7 | 1.18 | 46.15 | 0.57 | 0.33 | 0.81 | 18.2 |
| II | 64 | 64 | 54 | 2.78 | 15.63 | 0.82 | 0.73 | 0.89 | 0.0 | 59 | 64 | 55 | 1.39 | 14.06 | 0.87 | 0.80 | 0.94 | 8.5 |
| III | 3 | 3 | 2 | 0.24 | 33.33 | 0.66 | 0.23 | 1.00 | 0.0 | 4 | 4 | 3 | 0.29 | 25.00 | 0.75 | 0.41 | 1.00 | 0.00 |
| IV | 18 | 8 | 5 | 3.13 | 37.50 | 0.37 | 0.13 | 0.61 | -55.6 | 20 | 17 | 12 | 2.39 | 29.41 | 0.63 | 0.44 | 0.82 | -15.0 |
| V | 1 | 0 | 0 | 0.24 | - | 0.00 | 0.00 | 0.00 | -100.0 | 3 | 3 | 2 | 0.29 | 33.33 | 0.66 | 0.23 | 1.00 | 0.0 |
| VI | 10 | 10 | 6 | 0.97 | 40.00 | 0.59 | 0.33 | 0.85 | 0.0 | 9 | 9 | 7 | 0.58 | 22.22 | 0.77 | 0.56 | 0.99 | 0.0 |
| IX | 72 | 78 | 50 | 6.36 | 35.90 | 0.60 | 0.49 | 0.69 | 8.3 | 113 | 94 | 80 | 12.79 | 14.89 | 0.68 | 0.60 | 0.76 | -16.8 |
| X | 37 | 28 | 16 | 5.30 | 42.86 | 0.45 | 0.29 | 0.61 | -24.3 | 20 | 20 | 10 | 3.01 | 50.00 | 0.47 | 0.27 | 0.68 | 0.0 |
| XI | 20 | 18 | 13 | 1.72 | 27.78 | 0.67 | 0.49 | 0.84 | -10.0 | 20 | 17 | 11 | 2.69 | 35.29 | 0.57 | 0.37 | 0.77 | -15.0 |
| XII | 1 | 1 | 0 | 0.24 | 100.00 | 0.00 | -0.01 | 0.00 | 0.0 | 1 | 2 | 1 | 0.00 | 50.00 | 0.67 | 0.05 | 1.00 | 100.0 |
| XIII | 2 | 1 | 1 | 0.24 | 0.00 | 0.67 | 0.05 | 1.00 | -50.0 | 4 | 4 | 2 | 0.57 | 50.00 | 0.49 | 0.07 | 0.92 | 0.0 |
| XIV | 8 | 14 | 3 | 1.22 | 78.57 | 0.25 | 0.01 | 0.50 | 75.0 | 5 | 6 | 2 | 0.87 | 66.67 | 0.35 | -0.01 | 0.72 | 20.0 |
| XV | 0 | 0 | 0 | 0.00 | - | - | - | - | - | 2 | 0 | 0 | 0.57 | - | 0.00 | - | - | -100.0 |
| XVI | 5 | 7 | 4 | 0.24 | 42.86 | 0.66 | 0.35 | 0.97 | 40.0 | 2 | 3 | 2 | 0.00 | 33.33 | 0.80 | 0.41 | 1.00 | 50.0 |
| XVII | 6 | 6 | 4 | 0.48 | 33.33 | 0.66 | 0.35 | 0.97 | 0.0 | 3 | 3 | 2 | 0.29 | 33.33 | 0.66 | 0.23 | 1.00 | 0.0 |

The Bland-Altman diagram, ~~for~~ illustration the absolute difference in underlying CoD ~~in measurement~~ between the ~~"~~original~~"~~ and gold ~~"~~standard death certificates~~" DCs~~ ~~, is shown in Figure 1~~ (Fig 1), indicates a~~. The~~ mean difference ~~between "original" and "standard" DCs for the underlying CoD~~ was 0 (with limits of agreement: 3.5 to -3.5). T~~he comparison of the underlying CoD grouped by ICD-10 chapter, from the "original" and "standard" DCs, was t~~he variable plotted in the Bland-Altman diagram was the comparison of underlying CoD grouped by ICD-10 chapter from both the original and gold standard death certificates.

**Fig 1. Bland-Altman plot~~s.:~~ D~~d~~ifference between "original" and gold "standard" death certificate~~DC~~s for underlying CoD.**

# Discussion

This research study exploresis based on the complexities of the death certification process in Colombia–, offering valuable insights into the accuracy of Cause of Death (CoD) reportingand on the selection of cases in different territories at the national level. By examining a substancial sample size of 776 randomly selected death certificates fromThis study used the information recorded in medical records and health professionals trained in the preparation of DCs and the assignment of CoD. This provided a remarkable retrospective review.

Formatted: English (United States)

This research examined the accuracy of CoD in the DCs prepared in Colombia during 2021, the second year of the COVID-19 pandemic., our research employed a meticulous retrospective review, drawing upon information documented in medical records. The collaboration with health professionals adept in death certification preparation and CoD assignment added depth to our analysis. TFrom 776 randomly selected DCs, the overall agreement between both the "original" and gold "standard" death certificateDCs for the underlying CoD information, categorized according to the ICD-10 chapter, stood at a commendablewas 74%. Further stratification by gender revealed a slightly higher In men the conaccordance was 73% while in women was (75%) compared to men (73%). These results findings underscore demonstrate the nuance landscape of CoD estimation in Colombia, where both overestimation and underestimation contribute to the complexity of mortality statisticsfor some CoD in Colombia.

Comparing our results globally, tThe accuracy of the underlying underlying CoD reported by this researchin our study was highersurpassed figures than foundreported in Thailand (37%) (21) and Bangladesh (, where it was necessary to change the underlying CoD in 54%) of DCs (22), signifying a comparatively higher precision in Colombia. But However, it

fell short of the 81% ~~lower than~~ observed in Valparaíso and Metropolitan region in Chile ~~(81%)~~ (23). Disparities ~~ffering~~ in age ~~composition~~ demographics ~~of the population~~ and the unique challenges posed by ~~examination during~~ the ongoing pandemic ~~make limit~~ caution against direct comparisons, emphasizing ~~to these other studies included in~~ the the need for context-specific assessments~~observation period from the present analysis~~.

Our study highlighted notable overestimations and underestimations in specific CoD categories. ~~The agreement between the underlying CoD in “original” and “standard” DCs~~ H~~was h~~igh concordance was evident for "neoplasms" (Chapter II) and "codes for special situations (COVID-19)" (Chapter XXII), which ~~in 2021 represented~~collectively constituted 33% of ~~all~~ deaths in Colombia during 2021. ~~This study found~~O~~ o~~verestimation (a high percentages of false negatives and negative values in the CSMF) was identified for "circulatory system diseases" (Chapter IX); "pregnancy, childbirth and puerperium" (Chapter XV); "signs, symptoms, and poorly defined conditions" (Chapter XVIII) and "diseases of the respiratory system" (Chapter X), while ~~and~~ underestimation (a high percentages of CSMF) ~~of conditions such as~~ in "diseases of the genitourinary system" (Chapter XIV) among CoD.

The high level of concordance for deaths related to neoplasms, evidenced in the present research, coincides with an earlier study, ~~in~~ wh~~i~~ere~~ch~~ 93% of mortality from cancer in Colombia was well certified (16). A Mexican analysis showed that some types of cancer present a better concordance (24). The high concordance with COVID-19 diagnoses could be due to the standardized form that this new diagnosis was added to the list, as it includes information on the condition that started the causal chain of death (25).

The observed discrepancies in CoD assignment can be contextualized within the broader challenges of death certification in Colombia. The discrepancies observed in CoD assignment can be contextualized within the broader challenges of death certification in

Colombia. A substantial proportion of death certificates (79%) were prepared in health services by doctors, in some cases by health professionals with limited experience in this specific task or with a greater workload, which influences in not carefully reviewing the medical records to formulate the CoD sequence R~~In r~~egardi~~lation~~ng ~~to~~ deaths at home, ~~it was found that~~ some ~~of them do not have~~lack complete data in the medical records, experiencing~~;~~ ~~they have~~ greater difficulty in determining CoDs, and ~~the~~ family members ~~do~~may not provide enough information. In ~~cases of~~ rural areas, ~~the~~ certification is carried out by other untrained health professionals, ~~which generates~~~~intriooducing~~introducing possible errors in the certification process.(27)

Certification disparities exist between the certification of violent and non-violent deaths. Non-violent deaths are certified by the treating physician or other health professionals authorized to certify them, and violent deaths are certified by the forensic doctor (28). In ~~relation to DCs~~ terms of legal connotations of death certificates, it is true that the ~~the~~ certifier must recognize the potential effects that the logical elaboration of the CoD will have. Using certain CoDs may provide information that could be used to challenge, reduce, or deny life insurance benefits. Additionally, the ~~DC~~death certificate is rarely used as the sole source of information for legal or insurance purposes. ~~However~~Nevertheless, to reduce or avoid these situations, it is ~~important~~crucial to obtain correct and accurate information at all times (29).

Formatted: Indent: Left: -0.01", Right: -0.01"

This analysis has limitations. First, the determination of CoD was restricted to the information available in medical records, which may also ~~may~~ be imperfect. ~~-~~Second, in deaths ~~in~~ where~~ich~~ the clinical documentation was incomplete or in the event of a death that occurred at home, relatives or acquaintances of the deceased´s were interviewed, presenting possible recall bias. Third, ~~It can be considered that this study may have~~potential observer bias in the health professionals who analyzed the medical records

Formatted: Right: -0.01"

and completed the "standard" DC, due to the subjectivity of the observer when analyzing CoDs. A spin bias could also be considered due to a possible omission of data or an erroneous interpretation of the data, which depends on the interpretation that the observer has, generating an inaccuracy in the determination of the CoDs. However, the risk of bias is low because the health professionals who analyzed the medical records were trained in the DANE vital statistics course and were under permanent supervision in the information collection process. This study had methodological strengths such as the use of a gold standard death certificate, completed by trained health professionals. Additionally, it was based on solid criteria for the selection of cases in multiple jurisdcitionsjurisdictions at the national level, relying on complete medical records or complemented with information given by family members, representing a more precise analysis of the death certification process in Colombia. The differences between some of the CoDs identified between the original and gold standard death certificates are associated with the heterogeneity of the territories, where the death certification process varies depending on factors such as the lack of training of health professionals, incomplete medical records, difficulties in determining the CoDs, or internet access (15,24,28)..

In conclusion, our study sheds light on the commendablethe quality of the information on the underlying CoD information recorded in Colombian death certificateDCs, reflecting a 74% is considered good, considering the level of concordance between the official databases and with the gold "standard" deathtah certificateDC, was 74%. To facilitate and improve the process of DCsenhance ,the accuracy of the death certification process, we it is recommended to strengthenrobust educational and training programsinitiatives for the health professionals– ,, the development ofin charge of this task. Likewise, prepare comprehensive guidelines on the correctfor death certificate preparation, of the DCs; and carry outongoing monitoring in the health services to identify and address information

quality ~~problems~~issue~~, and propose alternatives for improvement~~. These measures are imperative for refining mortality statistics and ensuring the reliability of CoD data in the Colombian context.

## Acknowledgments

~~The authors wish to~~Special thanks to Dr. Richard Garfield of the Centers for Disease Control and Prevention for his valuable technical support ~~for~~ during the research development~~ of this research~~.

## References

1. AbouZahr C, Bratschi MW, Cercone E, Mangharam A, Savigny DD, Dincu I, et al. The COVID-19 Pandemic: Effects on Civil Registration of Births and Deaths and on Availability and Utility of Vital Events Data. Am J Public Health. 2021;111(6):1123-31. doi: 10.2105/AJPH.2021.306203.

2. AbouZahr C, De Savigny D, Mikkelsen L, Setel PW, Lozano R, Nichols E, et al. Civil registration and vital statistics: progress in the data revolution for counting and accountability. The Lancet. 2015;386(10001):1373-85. doi:10.1016/S0140-6736(15)60173-8.

3. AbouZahr C, Mathenge,Gloria, Brøndsted Sejersen T, Macfarlane SB. Civil Registration and Vital Statistics: A Unique Source of Data for Policy. The Palgrave Handbook of Global Health Data Methods for Policy and Practice. 2019;125-44.

4. Ling SM, Warner M, Anderson RN. Opportunities to Enhance the Utility of Cause of Death Information From Death Certificates. Am J Public Health. 2022;112(S1):S42-4. doi:10.2105/AJPH.2022.306722.

5. Merlin JS, Wachterman MW, Becker WC, Bohnert ASB. Serious Illness and Limitations of Death Certificate–Based Data: Urgent Lessons From the Opioid Epidemic. Am J Public Health. 2022;112(S1):S36-8. doi:10.2105/AJPH.2021.306409.

6. Centre of Excellence for CRVS Systems. Why CRVS systems are essential to achieving the Sustainable Development Goals. [Internet]. 2022. Available in: https://crvssystems.ca/index.php/

7. Filippatos G, Andriopoulos P, Panoutsopoulos G, Zyga S, Souliotis K, Gennimata V, et al. The quality of death certification practice in Greece. Hippokratia. 2016;20(1):19-25.

8. Hart JD, Sorchik R, Bo KS, Chowdhury HR, Gamage S, Joshi R, et al. Improving medical certification of cause of death: effective strategies and approaches based on experiences from the Data for Health Initiative. BMC Med. 2020;18(1):74. doi: 10.1186/s12916-020-01519-8.

9. França E, De Abreu DX, Rao C, Lopez AD. Evaluation of cause-of-death statistics for Brazil, 2002–2004. International Journal of Epidemiology. 2008;37(4):891-901. doi:10.1093/ije/dyn121.

10. Mikkelsen L, Phillips DE, AbouZahr C, Setel PW, De Savigny D, Lozano R, et al. A global assessment of civil registration and vital statistics systems: monitoring data quality and progress. The Lancet. 2015;386(10001):1395-406. doi: 10.1016/S0140-6736(15)60171-4.

11. Iburg KM, Mikkelsen L, Adair T, Lopez AD. Are cause of death data fit for purpose? evidence from 20 countries at different levels of socio-economic development. PLoS ONE. 2020;1:15. doi: 10.1371/journal.pone.0237539. doi: 10.1371/journal.pone.0237539.

12. OPS. Portal de indicadores básicos [Internet]. 2022. Available in: https://opendata.paho.org/es/indicadores-basicos/visualizaciones

13. Chaparro-Narváez PE, Jiménez-Serna MM, De La Hoz-Restrepo F. Identificación de los conocimientos de calidad de la información de mortalidad en Bogotá. Revista de salud pública. 2017;19(6):766-71. doi: 10.15446/rsap.V19n6.35377.

14. Cendales R, Pardo C. Quality of death certification in Colombia. Colomb Med. 2018;49(1):121-7. doi: 10.25100/cm.v49i1.3155.

15. Montalvo-Arce CA, Kelly-Forbes YS, Secretaría de Salud San Andrés. Evaluación de la calidad de los certificados de defunción del Hospital Amor de Patria, de San Andrés Islas, Colombia, en 2014. Revista Facultad Nacional de Salud Pública. 2018;36(3):61-70.

16. Cendales R, Pardo C. La calidad de certificación de la mortalidad en Colombia, 2002-2006. Revista de Salud Pública. 2011;13(2):229-38.

17. Rodríguez García J, Vega Landaeta AP, Jaramillo LF, Fernández Ortiz Y, Suarez Morales ZB, Vásquez Candia ME. Calidad en la certificación de mortalidad y natalidad en un hospital universitario, Colombia año 2014. Revista Gerencia y Políticas de Salud. 2019;18:1-25.

18. Chaparro Narváez PE. Calidad de la información de mortalidad en menores de un año en Bogotá durante 2005 [Internet]. [Bogotá, D.C]: Universidad Nacional de Colombia; 2013. Available in: https://repositorio.unal.edu.co/bitstream/handle/unal/20099/597600.2013.pdf?sequence=1&isAllowed=y

19. Landis JR, Koch GG. The measurement of observer agreement for categorical data. Biometrics.1977;33(1):159-74.

20. Rampatige R, Gamage S, Peiris S, Lopez AD. Assessing the Reliability of Causes of Death Reported by the Vital Registration System in Sri Lanka: Medical Records Review in Colombo. HIM J. 2013;42(3):20-8. doi: 10.1177/183335831304200302.

21. Washirasaksiri C, Raksasagulwong P, Chouriyagune C, Phisalprapa P, Srivanichakorn W. Accuracy and the factors influencing the accuracy of death certificates completed by first-year general practitioners in Thailand. BMC Health Serv Res. 2018;18(1):478. doi: 10.1186/s12913-018-3289-1.

22. Hazard RH, Chowdhury HR, Adair T, Ansar A, Quaiyum Rahman AM, Alam S, et al. The quality of medical death certification of cause of death in hospitals in rural Bangladesh: impact of introducing the International Form of Medical Certificate of Cause of Death. BMC Health Serv Res. 2017;17(1):688. doi: 10.1186/s12913-017-2628-y.

23. Antini C, Rajs D, Muñoz-Quezada MT, Mondaca BAL, Heiss G. Reliability of cause of death coding: an international comparison. Cad Saúde Pública. 2015;31(7):1473-82. doi: 10.1590/0102-311X00099814.

24. Hernández B, Ramírez-Villalobos D, Romero M, Gómez S, Atkinson C, Lozano R. Assessing quality of medical death certification: Concordance between gold standard diagnosis and underlying cause of death in selected Mexican hospitals. Popul Health Metrics. 2011;9(1):38. doi: 10.1186/1478-7954-9-38.

25. Armstrong D. The COVID-19 pandemic and cause of death. Sociology Health & Illness. 2021;43(7):1614-26. doi: 10.1111/1467-9566.13347.

26. Pritt BS, Hardin NJ, Richmond JA, Shapiro SL. Death Certification Errors at an Academic Institution. Archives of Pathology & Laboratory Medicine. 2005;129(11):1476-9. doi: 10.5858/2005-129-1476-DCEAAA.

~~26.~~

27.Vargas Lorenzo I. Barreras en el acceso a la atención en salud en modelos de competencia gestionada: un estudio de caso en Colombia. Universidad Autónoma de Barcelona. 2009. Available in: https://www.tdx.cat/handle/10803/4651

28. Juyal D, Kumar A, Pal S, Thaledi S, Jauhari S, Thawani V. Medical certification of cause of death during COVID-19 pandemic - a challenging scenario. J Family Med Prim Care. 2020 Dec 31;9(12):5896-5898. doi: 10.4103/jfmpc.jfmpc_1435_20. PMID: 33681015; PMCID: PMC7928126.

29. National Center for Health Statistics. Cause of Death and the Death Certificate Important Information for Physicians. Department of Health and Human Services. 1997.Available in: https://www.cdc.gov/nchs/nvss/writing-cod-statements/death_certification_problems.htm.

~~27.~~

~~28. Juyal D, Kumar A, Pal S, Thaledi S, Jauhari S, Thawani V. Medical certification of cause of death during COVID-19 pandemic - a challenging scenario. J Family Med Prim Care. 2020 Dec 31;9(12):5896-5898. doi: 10.4103/jfmpc.jfmpc_1435_20. PMID: 33681015; PMCID: PMC7928126.~~

**Formatted:** English (United States)

**Formatted:** Font: (Default) +Body (Calibri), Font color: Text 1

## DATABASE ACCURACY

All relevant data are within the manuscript and its Supporting Information files.

Bogotá, D.C, February 26th, 2024

Editors
**PLOS ONE**
Issue: rebuttal letter

Dear Editors,

We appreciate your review, general observations and comments from the reviewers that surely improve the quality of the present manuscript and allow us to discuss about the implications of our findings. Below you will find in blue the response to each point raised by the academic editor and reviewers.

**A. General comments**

1. Clear definition of PICO: Please restate the PICO structure research question clearly, focusing on the "reference gold standard CoD source" and the "original CoD source."

   The comment has been accepted. The research question was formulated with suggestions given and based on the PICO structure. The aim was adjusted in the “abstract” and “introduction” section (Ln 20-23 and Ln 82-85), according to the methodological design of the study as follows:

   The aim of the study was determined as follows: To estimate the accuracy of underlying CoD in the original death certificate, compared with a gold standard certificate based on information from clinical charts and relatives, in population deceased in Colombia during 2021”.

   Provide detailed selection criteria, sampling methods, and information about the study population. Revise all calculations thoroughly to ensure accurate comparisons and report the main outcomes according to the planned methods to prevent spin bias.

   Selection criteria, sampling methods and study population information were reviewed and adjusted in the “methods” section (Ln 87-102), to make clear the methodological design of the study.

   We reviewed the manuscript and result interpretation to assure that there is not spin bias, because the diagnoses of CoDs in the results, were established according to the national administrative department of statistics (DANE) and by an expert nosologist. So that, there is not subjectivity in the interpretation of the results.

2. Reproducible methods and research integrity compliance: To enhance reproducibility, provide full details about the CoD adjudicating committee and methods for determining causes of death.

We agreed that this subject should be explained better. Therefore, we provide details about the CoD adjudication committee and the methods for determining causes of death. It was adjusted and explained in the "methods" section (Ln 116-120), specifying that there was not a committee adjudicating of CoDs, but rather an expert nosologist did the codification of the CoDs.

3. Conclusions supported by data: Ensure that the discussion section addresses the implications of your research findings. Most importantly, suggest possible solutions that could improve accurate CoD adjudication, serving as a foundation for better healthcare policy and decision-making.

   We agreed with this comment related to the conclusions of the manuscript. Thus, we adjusted in the "discussion" section (Ln 299-304), considering the results of the research and suggesting some recommendations at the end of the manuscript.

4. Reporting guidelines and community standards: Comply with the IMRaD structure for scientific articles, including quality content items for the abstract section. It is highly recommended to follow the abstract and full-text STROBE statements for observational studies.

   The manuscript and abstract were adjusted with de IMRaD structure and STROBE statements, according to the guidelines requested by Plos One journal.

5. Article is written in an intelligible fashion and is in standard English: Perform a thorough English copyediting and coherence revision to ensure clear understanding of the research. Present manuscript sections, tables, and figures in an organized and appropriately classified manner.

   The writing of the manuscript in English was reviewed and adjusted. In addition, the tables and figures were reviewed, organized and adjusted, consistent with the topic of the research and according to the guidelines requested by Plos One journal.

6. Journal Requirements:

   All relevant data are within the manuscript and its Supporting Information files.
   Full ethical considerations were included in the "methods" section (Ln 139-152)

**General remarks**

7. The declared objective of the paper (line 82-85) is to compare the 'original certificate' with a more accurate information, so the "standard certificate" should be always used as the gold standard in the statistical analysis. Nevertheless, some indicators are calculated taking the original certificate as a reference. For instance, given a cause of death J, "false negatives" are certificates classified as J with the gold standard and classified differently by the "original certificate"

(which is under evaluation). “False positives” should be certificates in which the cause is other than J for the gold standard J for the “original certificate”. For example, for ICD10 chapter I, the false positives are 9 (from table 1), and the percentage of false positive 1.20. In the same way, “false negatives” are 8 and the percentage of false negative is 8/23*100= (34.8). (see table in the attached file). Nevertheless, in table 3 the percentages of false positives and false negatives are calculated using the original certificate as the gold standard. I suggest the authors to calculate the indicators using the standard certificate as the reference.

We agreed with the author's suggestion and founded an error in the formula and calculations in table 3. We modified the formula used in Table 3, using the gold standard certificate as the reference to adequately calculate false positives and negatives.

The indicator presented in line 114 seems to be calculated consistently with the objectives (standard certificate as gold standard) but the results presented in the last column of table 3 seem to have inverted signs.

The results had inverted signs, due to the error in the formula. We corrected it in Table 3 and explained it in the “results” section (Ln 187-196).

In the discussion, line 207, the authors report that there is an underestimation of (for instance) circulatory system disease, but in reality, the original certificate overestimates these causes compared to the gold standard statistics.
What the authors think about this? It would be better to calculate the indicators using the standard certificate as gold standard.

We did a thorough review verifying the calculations taking the standard certificate as gold standard and it was found that it really overestimates diseases of the circulatory system. The adjustment was made in the “discussion” section (Ln 250-255), clarifying the estimation of circulatory system disease.

8. I suggest to clarify the methods (Ln 95-106):
a. The trained health professionals, fill-in a new death form (part 1 and part 2 of the standard death certificate) or attribute a single cause-of-death?

Yes, trained healthcare professionals complete a new death form as a gold standard certificate based on the medical records and in a few cases the interviews to relatives (only in case the information in the medical records was incomplete), to build the causal chain of death and determine a final single cause of death. We clarified in the “methods” section (Ln 108-115),

b. Does the underlying cause codes for the original certificate correspond to those of official statistics for Colombia?

Yes, the underlying cause codes were determined according to the National Department of Statistics of Colombia and by an expert nosologist in this topic.

c. Is there a hierarchy of the medical documentation used for attributing the cause of death for the standard certificate? For deaths with more than one type of medical document (necropsy, clinical records, interview to relatives etc) only one is used for attributing the cause of death (as it appears from table 1)? In which order?

Yes, there is a hierarchy in medical documentation for attributing the cause of death for the standard certificate. First, the trained health professionals reviewed that the medical record was complete to determine the cause of death. Then they complemented with interviews to relatives, only if additional information was needed.

Only one is used for attributing the cause of death (as it appears from table 1)? In which order?

In general, only medical records were used to attribute the cause of death. In very few cases it was complemented with interviews to relatives in case information was missing in the medical records.

**Additional specific topics:**

9. I suggest to use the standard term "underlying cause" instead of "basic cause". We accepted the suggest of the reviewer and the term was adjusted by "underlying cause".

10. When ICD chapters are mentioned, I would suggest to use also the chapter number that would allow to find the results more easily in the tables.

The suggestion of the reviewer was accepted, and we included the chapter number.

11. Acronyms should be explained only once, the first time that are used (examples in line 28, CoD acronym described in the line above).

The acronyms were adjusted throughout the manuscript, according to the guidelines requested by Plos One Journal.

12. Ln 32-33. The sentence is not clear, what the author mean by "chart review" and "those who knew the medical history"?

The information was taken from medical records and when necessary, interviews were conducted to relatives or witnesses. The clarification was made in the abstract (Ln 27-29)

The terms are clear in the paper but they should be clear also in the abstract. Should it be "medical documentation" and "interviews to relatives"?

Same as previous answer, we clarified in the abstract that medical records were reviewed and when necessary, interviews to relatives were conducted to determine CoDs. The clarification was made in the abstract (Ln 27-29)

Ln 33: the certificate considered "standard" is the one based on the medical documentation and interviews?

Yes, the gold standard certificate is the one based on medical records. In a few cases the standard certificate was fill-in with information from interviews with family members or witnesses (only in case the information in the medical records was incomplete).

13.Ln 116-117, which variable and which points are plotted in the Bland-Altman diagram?

The Bland-Altman diagram, illustration the absolute difference in underlying CoD between the original and gold standard death certificates. The variable plotted in the Bland-Altman diagram was the comparison of underlying CoD grouped by ICD-10 chapter from both the original and gold standard death certificates. We explained in the "results" section (Ln 221-225).

14.Ln 124-130 and table 1. Is the sample analyzed (776 cases) representative of the total deaths in Colombia (in terms of age and sex)? A sentence regarding this aspect and on the possible distortion due to the loss of some cases from the desired sample would be useful.

Yes, it is representative of the total deaths in Colombia (in terms of age and sex). A nominal loss of 4% in the sample is considered a minimal reduction that did not introduce any distortion in the study findings. We explain it in the "results" section (Ln 154-157).

15.Ln 157-164. Confidence interval for Kappa should be used for comments and only robust values should be highlighted. For instance, results found for chapter "pregnancy childbirth and puerperium" are based only on two cases: it is not possible to evaluate false positives and Kappa is misleading. This should be taken into account.

We agreed with the reviewer and remove this information from the manuscript. We considered that it is not possible to determine the kappa statistic with only two cases of the chapter "pregnancy childbirth and puerperium".

16.Ln 167: The term "level of occurrence" is not clear. The table represents the Deaths by underlying cause according to the standard and original certificate and comparison of figures deriving from the two certificates.

We agreed with this comment and the title of table 3 was changed to: ¨Underlying CoD (ICD-10) agreement metrics between original and gold standard death certificates. Colombia, 2021¨ in the “results ‘section” (Ln 197-198).

17. Table 3. Last column (Change in cause – specific mortality fraction (%)): if the number of deaths for the original certificate is greater than for the standard, why the sign of the indicator is negative? According to the formula (line 114) I would expect a positive sign (and vice-versa for positive values).

Regarding this comment, we reviewed and founded an error in the formula in table 3. We adjusted it including the last column as % specific mortality fraction (Ln 200-201). In consequence results changed and were explained according to the findings.

18.Ln 171-177: This part presents the results for men and women (is it correct?), but it seems a repetition of the above comment. I suggest to mention that for most causes the results are comparable in the two genders and successively to focus only on chapters where there are differences.

We agreed with the reviewer's suggestion, and therefore the text was adjusted in the “results” section (Ln 203-217) focusing in the differences between genders.

19. Ln 177: Fig 1 is a table, it should be considered table 4.

We adjusted the table 4 in the “results” section (Ln 218-220), according to the guidelines of tables and figures requested by Plos One journal.

20.Ln 182: The term validity in this context is not clear (the description of this table should be similar to table 2 which has similar content).

We agreed with the reviewer and adjusted the table 2 in the “results” section (Ln 176-177). We changed the term validity to: “Cross tabulation of underlying CoD (ICD-10) in original and gold standard death certificates. Colombia, 2021”.

21.Figure 1 (should be table 4): the columns’ titles for women are different than for men and they have different order.

We agreed with the reviewer and the table 4 was adjusted in the “results” section (Ln 218-220). We corrected the men's and women's columns.

22.Ln 207-209: The authors report underestimation for ‘circulatory system disease’ but the results seem to show that the original death certificate overestimates them. The same occurs for ‘diseases of respiratory system’. This term should be used when the original death certificate, which is under evaluation, detects more cases than the standard. The use of the term underestimation considering the false negatives is misleading. I would suggest to use the CSMF, which is calculated but little used in comments.

We agreed with the comment of the reviewer and adjusted in the “discussion” section (Ln 247-255), considering that it was an overestimation of diseases of the respiratory system in the original certificate. We also agreed using the CMSF in the described discussion paragraph.

23.Ln 210: The result about ‘pregnancy, childbirth and puerperium’ is very counterintuitive, since death certificates are often considered to underestimate maternal mortality, while examining additional documentation should allow to detect more accurately the cases. What is the authors’ opinion on this result?

We considered removing this information from the manuscript, because it was not appropriate to determine results with few cases from this chapter, as we explained in comment No.15. Additionally, these cases do not have complete information in the medical records, and it was not possible to determine a precise cause of death.

In addition, it is surprising that there are 7 cases classified in chapter XVII (Symptoms and signs) by the standard certificates which is based on additional documentation. Is there some explanation for these cases?

In Colombia, analyzes of maternal deaths are carried out to conclude whether the death corresponded to a pregnant woman according to the definitions used in public health surveillance. For these seven cases, the sources of information (medical records) were not complete and did not contribute to the causal chain of death. For this reason, the basic cause of death could not be related in chapter XV (pregnancy, childbirth and puerperium).

24.Ln 216-218: Could authors rephrase this sentence? It is not very clear.

Yes, we can rephrase this sentence. We left it as follows: “A substantial proportion of death certificates (79%) were prepared in health services by doctors, in some cases by health professionals with limited experience in this specific task or with a greater workload, which influences in not carefully reviewing the medical records to formulate the CoD sequence.”

We adjusted the sentence in the “discussion” section (Ln 263-266).

25.Ln 224: Are there differences for deaths occurred in hospital compared to other places?

Yes, there are differences between deaths occurred in hospital compared to other places. We added a paragraph explaining this difference with its respective bibliographic reference. We added a paragraph with this subject in the “discussion” section (Ln 262-273).

Are there differences for source of determination of the cause of death? If possible, some comments could be provided in the discussion (even if not provided in the results).

Yes, there are differences between the source of determination of the cause of death, depending on if the death occurs in the hospital or other places. We explained this subject in the “discussion” section (Ln 262-273).

**Major points:**

26. The article essentially argues that death certificates are incomplete and imprecise. The apparent aim of the article is to estimate the accuracy of this information in some way.

    Yes, we agreed that the aim of this research is to estimate accuracy. In that way we adjusted the title and aim of the manuscript according with this comment.

    In the methods section, it describes how this was achieved, using statistical measures such as kappa and clinical record data for these comparisons.

    Yes, we considered changing the term "validity" to "accuracy" in the title and aim of the manuscript, according to the methodological design and the use of kappa statistic.

    Moreover, it excessively dwells on situational aspects and also introduces references that appear disjointed, hindering the reader's understanding of whether these are issues pertinent to countries similar to Colombia, or if they represent a standard that should be adopted.

    We agreed with the reviewer comment. We reviewed the introduction which was very long and excessively dwells on situational aspects, so it was adjusted to make it more concise and coherent with the aim of the study. The referenced bibliography refers to a statistical calculation used in several studies worldwide and to a topic related to the certification of deaths process that happens in all countries.

27. Informed Consent and Access to Records: One of the significant methodological issues is the lack of clarity around consent for accessing clinical records and comparing them with death registrations. It is not stated whether consent was obtained from the deceased's family or from the physician who completed the death certificate. Clarification on the consent process is crucial to address potential ethical concerns.

    In this regard, on April 27, 2022, the Committee on Ethics and Research Methodologies of the National Institute of Health (In Spanish Committee on Ethics and Research Methodologies - CEMIN) stated that “the National Institute of Health through the National Health Observatory , can act as a health authority and within

the framework of its missionary functions request from the respective Health Providing Institutions (IPS) access to medical records in accordance with current regulations (Art. 20, Law 57 of 1985; Art. 38 , literal d, Law 23 of 1981; Art. 14, paragraph 3 of Resolution 1995 of 1999).

Then, on July 19, 2022, the same Committee authorized verbal interviews through telephone calls to the relatives or legal representatives of the deceased to complement the missing information in the medical record. It was recommended to "record the telephone call from the beginning, with the prior acceptance of the interlocutor, in such a way that their verbal informed consent was recorded to provide the information required for the exclusive purpose of this activity."

We adjusted ethics considerations in ´methods ‘section (Ln 139-152) with a clearer explanation of this subject.

28. Legal Implications of Findings: The article does not discuss how it will address legal issues arising from its findings. Given the association of death diagnoses with matters such as life insurance, as mentioned in the document, this oversight could lead to legal disputes involving the families of the subjects and the healthcare professionals involved.

    We agreed with this point, and we added an additional paragraph in the manuscript explaining the analysis of the legal implications.

    We added this paragraph in "discussion" section (Ln: 273-279), in the following way: "In terms of legal connotations of death certificates, it is true that the certifier must recognize the potential effects that the logical elaboration of the CoD will have. Using certain CoDs may provide information that could be used to challenge, reduce, or deny life insurance benefits. Additionally, the death certificate is rarely used as the sole source of information for legal or insurance purposes. Nevertheless, to reduce or avoid these situations, it is crucial to obtain correct and accurate information at all times."

29. Qualifications of Data Reviewers: The educational background of individuals reviewing the clinical histories is not disclosed. Knowing their qualifications is imperative to assess the credibility of the data interpretation.

    Yes, we agreed with this comment and added this information in "methods" section (Ln 103-107), including the educational background of health professionals and their qualifications.

30. Data Anonymization and Interviews: There are significant doubts about the anonymization process, especially since family members or caregivers were interviewed. It is unclear if informed consent was obtained for these interviews and whether the participants were made aware of the potential risks.

We agreed that the anonymization process of the study should be clarified in the manuscript, especially in interviews to relatives. Ethics considerations were adjusted in “methods” section (Ln 146-152).

31. Use of Kappa Statistic: The application of the kappa statistic does not inherently validate the findings. The methodological design seems to focus on measuring agreement or discrepancies but does not support the claimed validity of the results.

    According with the comment, we changed the tittle and aim of the manuscript because this is a study of concordance and not validity. The concordance study supports the use of the Kappa statistic.

32. Methodological Framework and Statistical Sufficiency: There is no methodological framework explaining why using a kappa statistic is sufficient for the validity mentioned. Further explanation is needed to justify this approach.

    According to the previous response, we made explicit the scope of the analysis, therefore, it was specified that it is a concordance study, adjusting the title and aim of the manuscript.

**Minor Concerns:**

33. The definitions of “false positive” and “false negative” are not provided in either the introduction or the methods section, which could lead to ambiguity in interpreting the study's results.

    We agreed with this comment and included these definitions in “methods” section (Ln 124-125).
