# Supplementary material for: Accuracy of information on the underlying cause of death: An analysis in Colombia during the COVID-19 pandemic in 2021
Source: PLoS One. 2025 May 20;20(5):e0320466. doi: 10.1371/journal.pone.0320466 (PMC12092014; doi:10.1371/journal.pone.0320466)
Supplement: Attachment — Submitted filename: PONE-D-23-33548R1-Review.pdf [file pone.0320466.s008.pdf]

PONE-D-23-33548R1

Accuracy of Information on the Underlying Cause of Death: An Analysis in Colombia during the COVID-19 Pandemic in 2021

Journal: PLOS ONE

Review due date: Mar. 19, 2024

Thank you for the opportunity to review the revised manuscript titled "Accuracy of Information on the Underlying Cause of Death: An Analysis in Colombia during the COVID-19 Pandemic in 2021." Despite the revisions, there remain several areas requiring further attention to strengthen the study:

Major Points:

Originality and comparative analysis: The article's novelty is not clearly established. A comparative analysis with previous years or subsequent data (2019, 2020, 2022) would provide context to evaluate changes in accuracy over time, and specially the relation/effect of COVID-19 in registries precision.

Methodological clarity and ethical considerations: The temporal scope of the mortality records reviewed remains unclear (It needs to be precised). Additionally, the article must address whether informed consents were secured for the review of medical records and how potential biases during the data review process were mitigated. It is also critical to clarify the anonymization process in methodology and ethical considerations involved, especially in relation to the conducted interviews (include questions applied to participants in appendices).

Sampling and data source engagement with DANE: The sampling method from the 326,833 certificates and the decision to utilize data from the INS instead of DANE

requires clarification. It's essential to understand whether the co-authors engaged with DANE regarding the study. Notably, the lack of substantial references from DANE or local studies to corroborate the significance of this issue is a concern, with only reference number 13 addressing local context, and references 14 to 18 providing some local insight. A stronger consideration of DANE as the official data source is necessary.

Statistical analysis and methodology: Concerns arise regarding the calculation of false positives and negatives, which need to be clarified (specify whether these measures support the analysis). The methodological rationale behind using kappa statistics solely for establishing validity is insufficiently explained.

Representativeness and underestimation: The claims of underestimation of certain causes of death and the representativeness of these within the sample need substantiation, especially for rare events (additionally evaluate how to operate kappa in circumstances where there are few observations).

Minor points

Definition of "gold standard certificate": The term "gold standard certificate" is used without explanation (could be difficult for reader, use a unique term). A clear definition is necessary to avoid confusion and ensure reader comprehension.

Interviews and recording custodianship: The use of telephone interviews requires clarification on consent, custodianship of the recordings, contingency plans for non-participation, and the refusal rates, as well as transparency about the questions asked (include instrument in appendices).
Inclusion of interview data in results: The application of interview data within the results is not demonstrated, which raises questions about its influence on the study's conclusions.

Decimals in sample size: The presence of decimals in sample size in Table 1 raises questions about the data presentation, which typically requires whole numbers.

Discussion of accuracy vs concordance: The conclusions focus on concordance rather than accuracy, which is a central theme. The impact of the pandemic on the study's validity should be assessed, especially since the title starts with COVID-19, implying a primary concern with the pandemic's influence on data precision requiring to analyze additional years.

Bibliographical Strength and Relevance: The bibliographic support for key arguments, particularly regarding the relevance of accuracy in death certification to public health decision-making, is insufficient. The introduction would benefit from robust, primary sources, and all references should comply with citation standards, including DOI where applicable.
