# Supplementary material for: Accuracy of information on the underlying cause of death: An analysis in Colombia during the COVID-19 pandemic in 2021
Source: PLoS One. 2025 May 20;20(5):e0320466. doi: 10.1371/journal.pone.0320466 (PMC12092014; doi:10.1371/journal.pone.0320466)
Supplement: Attachment — Submitted filename: PONE-D-23-33548R3 Review Jul 28 2024.pdf [file pone.0320466.s010.pdf]

PONE-D-23-33548R2

Accuracy of Information on the Underlying Cause of Death: An Analysis in Colombia during the COVID-19 Pandemic in 2021

Journal: PLOS ONE

Review due date: Jul. 28, 2024

Thank you for the opportunity to review the revised manuscript titled "Accuracy of Information on the Underlying Cause of Death: An Analysis in Colombia during the COVID-19 Pandemic in 2021." Despite the revisions, there remain several areas requiring further attention to strengthen the study:

Major Points:

Impact of the COVID-19 Pandemic on Accuracy:

Observation: It is not clear if the accuracy levels observed were influenced by the pandemic. Adding "during the pandemic" to the title does not sufficiently address this issue.

Recommendation: Elaborate on how the COVID-19 pandemic might have affected the accuracy of death certificates. Consider including a comparative analysis with data from non-pandemic years to assess whether the observed accuracy levels are typical or if they were significantly impacted by the pandemic. This would provide valuable context and enhance the relevance of the study's findings.

Historical context:

- Observation: Is the statement about the knowledge of causes of death before 1991 in Colombia accurate?
- Lines 86-89: Provide context on the historical accuracy of death cause determination in Colombia and its comparison with international registries.

Justification and bibliographic support:

- Observation: The assertion about INS's role needs bibliographic support.
- Lines 100-105: Explain why INS is interested in evaluating DANE's data and how telephone interviews contribute to data accuracy. Mention the role of kappa statistics in this context.

Data reconstruction:

- Observation: Were the data reconstructed by researchers or provided by DANE?
- Lines 115-118: Clarify if the data were reconstructed by the researchers or consolidated by DANE, and if mentioning another institute in this line is necessary.

Custodianship of medical records:

- Observation: Who holds the medical records in Colombia? How were they accessed for this study?
- Lines 120-121: Explain who is the custodian of medical records and how the researchers accessed them for the study.

Data custodianship upon death:

- Observation: Who is the custodian of medical records upon death in Colombia?
- Lines 129-130: Clarify custodianship of medical records (IPS, EPS, DANE, or INS) and the process followed in this study.

Interview data application:

- Observation: How were the interviews conducted, and how was the information interpreted?
- Lines 138-141: Detail the process of interpreting interview data and its application in determining causes of death.
- Observation: Can replacements from interviews be identified in Table 3?
- Line 234: Indicate if interview-derived data replacements are identifiable in Table 3.

Change in cause specific mortality fraction:

- Observation: Explain the purpose and values of this metric.
- Lines 251, 257, 265: Clarify the significance of "change in cause specific mortality fraction" and why there are extreme and negative values.

Discussion relevance:

- Observation: Certain parts of the discussion do not contribute to the manuscript.
- Lines 291-292: Remove or rephrase parts of the discussion that do not add value to the manuscript.

Analysis focus:

- Observation: Is the focus on transcription accuracy rather than the accuracy of the records themselves?
- Lines 294-295: Clarify if the analysis is about transcription accuracy or the accuracy of the death records themselves.

Reliance on family reports:

- Observation: Does relying on family reports undermine the medical expert's role?
- Lines 294-295, 306-309: Address how family reports are interpreted and ensure that the role of medical experts is not undermined.

Interview information collection:

- Observation: How was information from telephone interviews collected and verified?
- Lines 339-341: Detail the process of collecting and verifying information from telephone interviews, considering potential biases (educational level of the interviewee).

Territorial analysis:

- Observation: There is no analysis of territorial differences in record accuracy.
- Lines 365-369: Include an analysis of territorial differences in the accuracy of death records and the impact of training, medical record completeness, and internet access.

Minor points:

Abstract clarity:

- Observation: In the abstract, mentioning "original death certificate" is unclear. What does "original" refer to— the original medical record?

- Line 20: Clarify whether the "original death certificate" refers to the original medical records or another source.

Bibliographical support:
- Observation: Reference number 13 does not support the discussion in lines 77-81.
- Lines 77-81: Ensure that references are correctly aligned with the content they support.

Provision of documents:
- Observation: Was the data from DANE provided registry by registry or as a complete database?
- Lines 24-26: Specify if the 326,833 original certificates were provided as a complete database by the National Department of Statistics (DANE). Why if it was provided a database of 326.833 registries, it was not used the whole data?

Terminology confusion:
- Observation: Using "original" and "standard" is confusing. Can these terms be replaced by "source" or similar terms?
- Lines 27-29: Replace "original" and "standard" with clearer terms like "source" or "database".

Terminology in results:
- Observation: It is difficult to interpret which is the original and which is the standard certificate.

Entity mentions:
- Observation: There are inconsistencies in mentioning the entities.
- Lines 25-26 and 86: Ensure consistent use of entity names throughout the manuscript. Confirm if "National Department of Statistics" and "National Statistics Institute (DANE)" refer to the same entity.
- Lines 33-34: Ensure clarity in distinguishing between original and gold standard certificates.

Expected proportion:
- Observation: What does "expected proportion" refer to?
- Line 126: Define "expected proportion".

Temporal scope:
- Observation: The exact period of deaths analyzed can aid in understanding sample randomness.
- Lines 132-133: Provide the exact dates of the deaths analyzed to understand the sample's randomness better. At least the first date of death and the last date of death considered.

Terminology in methods:
- Observation: Clarify the use of "standard," "original," and "medical record death."
- Lines 134-141: Ensure consistent and clear terminology to differentiate between data sources.

Terminology in tables:
- Observation: Adjust the column name "standard" to "gold standard" in Table 4.
- Line 251: Rename the column in Table 4 to avoid confusion.

Ensure proper case use in formulas and table headers.
Use consistent terminology throughout the manuscript to avoid confusion.
