# Supplementary material for: Accuracy of information on the underlying cause of death: An analysis in Colombia during the COVID-19 pandemic in 2021
Source: PLoS One. 2025 May 20;20(5):e0320466. doi: 10.1371/journal.pone.0320466 (PMC12092014; doi:10.1371/journal.pone.0320466)
Supplement: Attachment — Submitted filename: renamed_26069.docx [file pone.0320466.s004.docx]

PONE-D-23-33548

Validity of the information on the basic cause of death: an analysis in Colombia during the COVID-19 pandemic in 2021

Journal: PLOS ONE

Review due date: Nov. 19, 2023

The article essentially argues that death certificates are incomplete and imprecise. The apparent aim of the article is to estimate the accuracy of this information in some way. In the methods section, it describes how this was achieved, using statistical measures such as kappa and clinical record data for these comparisons. It is noteworthy that the background describes the available information in a confusing manner. Moreover, it excessively dwells on situational aspects and also introduces references that appear disjointed, hindering the reader's understanding of whether these are issues pertinent to countries similar to Colombia, or if they represent a standard that should be adopted.

Major points:

1. Informed Consent and Access to Records: One of the significant methodological issues is the lack of clarity around consent for accessing clinical records and comparing them with death registrations. It is not stated whether consent was obtained from the deceased's family or from the physician who completed the death certificate. Clarification on the consent process is crucial to address potential ethical concerns.
2. Legal Implications of Findings: The article does not discuss how it will address legal issues arising from its findings. Given the association of death diagnoses with matters such as life insurance, as mentioned in the document, this oversight could lead to legal disputes involving the families of the subjects and the healthcare professionals involved.
3. Qualifications of Data Reviewers: The educational background of individuals reviewing the clinical histories is not disclosed. Knowing their qualifications is imperative to assess the credibility of the data interpretation.
4. Data Anonymization and Interviews: There are significant doubts about the anonymization process, especially since family members or caregivers were interviewed. It is unclear if informed consent was obtained for these interviews and whether the participants were made aware of the potential risks.
5. Use of Kappa Statistic: The application of the kappa statistic does not inherently validate the findings. The methodological design seems to focus on measuring agreement or discrepancies but does not support the claimed validity of the results.
6. Methodological Framework and Statistical Sufficiency: There is no methodological framework explaining why using a kappa statistic is sufficient for the validity mentioned. Further explanation is needed to justify this approach.

Minor Concerns:

The definitions of 'false positive' and 'false negative' are not provided in either the introduction or the methods section, which could lead to ambiguity in interpreting the study's results.

In conclusion, while the research addresses an important issue, these methodological concerns need addressing to enhance the study's robustness and ethical compliance.
