# Supplementary material for: Accuracy of information on the underlying cause of death: An analysis in Colombia during the COVID-19 pandemic in 2021
Source: PLoS One. 2025 May 20;20(5):e0320466. doi: 10.1371/journal.pone.0320466 (PMC12092014; doi:10.1371/journal.pone.0320466)
Supplement: Attachment — Submitted filename: ValidityCauseDeathAuthors.docx [file pone.0320466.s005.docx]

**Validity of the information on the basic cause of death: an analysis in Colombia during the Covid-19 pandemic**

Dear authors, the paper is interesting and analyzes a crucial problem. Nevertheless, I suggest you an additional effort to better clarify some aspects of the methods and to improve the consistency of data analysis.

General remarks

1. The declared objective of the paper (line 82-85) is to compare the ‘original certificate’ with a more accurate information, so the “standard certificate” should be always used as the gold standard in the statistical analysis. Nevertheless, some indicators are calculated taking the original certificate as a reference. For instance, given a cause of death J, “false negatives” are certificates classified as J with the gold standard and classified differently by the “original certificate” (which is under evaluation). “False positives” should be certificates in which the cause is other than J for the gold standard J for the “original certificate”. For example, for ICD10 chapter I, the false positives are 9 (from table 1), and the percentage of false positive 1.20. In the same way, “false negatives” are 8 and the percentage of false negative is 8/23*100= (34.8). (see table below). Nevertheless, in table 3 the percentages of false positives and false negatives are calculated using the original certificate as the gold standard. I suggest the authors to calculate the indicators using the standard certificate as the reference.

Agreement and disagreement for chapter 1

|  |  | Gold standard | | |
| --- | --- | --- | --- | --- |
| Original certificate |  | Yes | No |  |
|  | Yes | 15 | 9 | 24 |
|  | No | 8 | 744 | 752 |
|  |  | 23 | 753 | 776 |

Percentage of false positive = 9/753 *100= 1,20

Percentage of false negative = 8/23 *100= 34,78

The indicator presented in line 114 seems to be calculated consistently with the objectives (standard certificate as gold standard) but the results presented in the last column of table 3 seem to have inverted signs.

In the discussion, line 207, the authors report that there is an underestimation of (for instance) circulatory system disease, but in reality, the original certificate overestimates these causes compared to the gold standard statistics.

What the authors think about this? It would be better to calculate the indicators using the standard certificate as gold standard.

2. I suggest to clarify the methods (Ln 95-106):
   a. The trained health professionals, fill-in a new death form (part 1 and part 2 of the standard death certificate) or attribute a single cause-of-death?
   b. Does the underlying cause codes for the original certificate correspond to those of official statistics for Colombia?
   c. Is there a hierarchy of the medical documentation used for attributing the cause of death for the standard certificate? For deaths with more than one type of medical document (necropsy, clinical records, interview to relatives etc) only one is used for attributing the cause of death (as it appears from table 1)? In which order?

Additional specific topics:

1. I suggest to use the standard term “underlying cause” instead of “basic cause”.
2. When ICD chapters are mentioned, I would suggest to use also the chapter number, that would allow to find the results more easily in the tables.
3. Acronyms should be explained only once, the first time that are used (examples in line 28, CoD acronym described in the line above).
4. Ln 32-33. The sentence is not clear, what the author mean by “chart review” and “those who knew the medical history”? The terms are clear in the paper but they should be clear also in the abstract. Should it be “medical documentation” and “interviews to relatives”? Ln 33: the certificate considered “standard” is the one based on the medical documentation and interviews?
5. Ln 116-117, which variable and which points are plotted in the Bland-Altman diagram?
6. Ln 124-130 and table 1. Is the sample analyzed (776 cases) representative of the total deaths in Colombia (in terms of age and sex)? A sentence regarding this aspect and on the possible distortion due to the loss of some cases from the desired sample would be useful.
7. Ln 157-164. Confidence interval for Kappa should be used for comments and only robust values should be highlighted. For instance, results found for chapter “pregnancy childbirth and puerperium” are based only on two cases: it is not possible to evaluate false positives and Kappa is misleading. This should be taken into account.
8. Ln 167: The term “level of occurrence” is not clear. The table represents the Deaths by underlying cause according to the standard and original certificate and comparison of figures deriving from the two certificates.
9. Table 3. Last column (Change in cause – specific mortality fraction (%)): if the number of deaths for the original certificate is greater than for the standard, why the sign of the indicator is negative? According to the formula (line 114) I would expect a positive sign (and vice-versa for positive values).
10. Ln 171-177: This part presents the results for men and women (is it correct?), but it seems a repetition of the above comment. I suggest to mention that for most causes the results are comparable in the two genders and successively to focus only on chapters where there are differences.
11. Ln 177: Fig 1 is a table, it should be considered table 4.
12. Ln 182: The term validity in this context is not clear (the description of this table should be similar to table 2 which has similar content).
13. Figure 1 (should be table 4): the columns’ titles for women are different than for men and they have different order.
14. Ln 207-209: The authors report underestimation for ‘circulatory system disease’ but the results seem to show that the original death certificate overestimates them. The same occurs for ‘diseases of respiratory system’. This term should be used when the original death certificate, which is under evaluation, detects more cases than the standard. The use of the term underestimation considering the false negatives is misleading. I would suggest to use the CSMF, which is calculated but little used in comments.
15. Ln 210: The result about ‘pregnancy, childbirth and puerperium’ is very counterintuitive, since death certificates are often considered to underestimate maternal mortality, while examining additional documentation should allow to detect more accurately the cases. What is the authors’ opinion on this result? In addition, it is surprising that there are 7 cases classified in chapter XVII (Symptoms and signs) by the standard certificates which is based on additional documentation. Is there some explanation for these cases?
16. Ln 216-218: Could authors rephrase this sentence? It is not very clear.
17. Ln 224: Are there differences for deaths occurred in hospital compared to other places? Are there differences for source of determination of the cause of death? If possible, some comments could be provided in the discussion (even if not provided in the results).
