# Supplementary material for: Accuracy of information on the underlying cause of death: An analysis in Colombia during the COVID-19 pandemic in 2021
Source: PLoS One. 2025 May 20;20(5):e0320466. doi: 10.1371/journal.pone.0320466 (PMC12092014; doi:10.1371/journal.pone.0320466)
Supplement: Attachment — Submitted filename: Response to reviewers.docx [file pone.0320466.s006.docx]

Bogotá, D.C, February 26^th^, 2024

Editors

**PLOS ONE**

Issue: rebuttal letter

Dear Editors,

We appreciate your review, general observations and comments from the reviewers that surely improve the quality of the present manuscript and allow us to discuss about the implications of our findings. Below you will find in blue the response to each point raised by the academic editor and reviewers.

A. **General comments**
1. Clear definition of PICO: Please restate the PICO structure research question clearly, focusing on the "reference gold standard CoD source" and the "original CoD source."

The comment has been accepted. The research question was formulated with suggestions given and based on the PICO structure. The aim was adjusted in the “abstract” and “introduction” section (Ln 20-23 and Ln 82-85), according to the methodological design of the study as follows:

The aim of the study was determined as follows: To estimate the accuracy of underlying CoD in the original death certificate, compared with a gold standard certificate based on information from clinical charts and relatives, in population deceased in Colombia during 2021”.

Provide detailed selection criteria, sampling methods, and information about the study population. Revise all calculations thoroughly to ensure accurate comparisons and report the main outcomes according to the planned methods to prevent spin bias.

Selection criteria, sampling methods and study population information were reviewed and adjusted in the “methods” section (Ln 87-102), to make clear the methodological design of the study.

We reviewed the manuscript and result interpretation to assure that there is not spin bias, because the diagnoses of CoDs in the results, were established according to the national administrative department of statistics (DANE) and by an expert nosologist. So that, there is not subjectivity in the interpretation of the results.

2. Reproducible methods and research integrity compliance: To enhance reproducibility, provide full details about the CoD adjudicating committee and methods for determining causes of death.

We agreed that this subject should be explained better. Therefore, we provide details about the CoD adjudication committee and the methods for determining causes of death. It was adjusted and explained in the “methods” section (Ln 116-120), specifying that there was not a committee adjudicating of CoDs, but rather an expert nosologist did the codification of the CoDs.

3. Conclusions supported by data: Ensure that the discussion section addresses the implications of your research findings. Most importantly, suggest possible solutions that could improve accurate CoD adjudication, serving as a foundation for better healthcare policy and decision-making.

We agreed with this comment related to the conclusions of the manuscript. Thus, we adjusted in the “discussion” section (Ln 299-304), considering the results of the research and suggesting some recommendations at the end of the manuscript.

4. Reporting guidelines and community standards: Comply with the IMRaD structure for scientific articles, including quality content items for the abstract section. It is highly recommended to follow the abstract and full-text STROBE statements for observational studies.

The manuscript and abstract were adjusted with de IMRaD structure and STROBE statements, according to the guidelines requested by Plos One journal.

5. Article is written in an intelligible fashion and is in standard English: Perform a thorough English copyediting and coherence revision to ensure clear understanding of the research. Present manuscript sections, tables, and figures in an organized and appropriately classified manner.

The writing of the manuscript in English was reviewed and adjusted. In addition, the tables and figures were reviewed, organized and adjusted, consistent with the topic of the research and according to the guidelines requested by Plos One journal.

6. Journal Requirements:

All relevant data are within the manuscript and its Supporting Information files.

Full ethical considerations were included in the “methods” section (Ln 139-152)

**General remarks**

7. The declared objective of the paper (line 82-85) is to compare the ‘original certificate’ with a more accurate information, so the “standard certificate” should be always used as the gold standard in the statistical analysis. Nevertheless, some indicators are calculated taking the original certificate as a reference. For instance, given a cause of death J, “false negatives” are certificates classified as J with the gold standard and classified differently by the “original certificate” (which is under evaluation). “False positives” should be certificates in which the cause is other than J for the gold standard J for the “original certificate”. For example, for ICD10 chapter I, the false positives are 9 (from table 1), and the percentage of false positive 1.20. In the same way, “false negatives” are 8 and the percentage of false negative is 8/23*100= (34.8). (see table in the attached file). Nevertheless, in table 3 the percentages of false positives and false negatives are calculated using the original certificate as the gold standard. I suggest the authors to calculate the indicators using the standard certificate as the reference.

We agreed with the author's suggestion and founded an error in the formula and calculations in table 3. We modified the formula used in Table 3, using the gold standard certificate as the reference to adequately calculate false positives and negatives.

The indicator presented in line 114 seems to be calculated consistently with the objectives (standard certificate as gold standard) but the results presented in the last column of table 3 seem to have inverted signs.

The results had inverted signs, due to the error in the formula. We corrected it in Table 3 and explained it in the “results” section (Ln 187-196).

In the discussion, line 207, the authors report that there is an underestimation of (for instance) circulatory system disease, but in reality, the original certificate overestimates these causes compared to the gold standard statistics.

What the authors think about this? It would be better to calculate the indicators using the standard certificate as gold standard.

We did a thorough review verifying the calculations taking the standard certificate as gold standard and it was found that it really overestimates diseases of the circulatory system. The adjustment was made in the “discussion” section (Ln 250-255), clarifying the estimation of circulatory system disease.

8. I suggest to clarify the methods (Ln 95-106):

a. The trained health professionals, fill-in a new death form (part 1 and part 2 of the standard death certificate) or attribute a single cause-of-death?

Yes, trained healthcare professionals complete a new death form as a gold standard certificate based on the medical records and in a few cases the interviews to relatives (only in case the information in the medical records was incomplete), to build the causal chain of death and determine a final single cause of death. We clarified in the “methods” section (Ln 108-115),

b. Does the underlying cause codes for the original certificate correspond to those of official statistics for Colombia?

Yes, the underlying cause codes were determined according to the National Department of Statistics of Colombia and by an expert nosologist in this topic.

c. Is there a hierarchy of the medical documentation used for attributing the cause of death for the standard certificate? For deaths with more than one type of medical document (necropsy, clinical records, interview to relatives etc) only one is used for attributing the cause of death (as it appears from table 1)? In which order?

Yes, there is a hierarchy in medical documentation for attributing the cause of death for the standard certificate. First, the trained health professionals reviewed that the medical record was complete to determine the cause of death. Then they complemented with interviews to relatives, only if additional information was needed.

Only one is used for attributing the cause of death (as it appears from table 1)? In which order?

In general, only medical records were used to attribute the cause of death. In very few cases it was complemented with interviews to relatives in case information was missing in the medical records.

**Additional specific topics:**

9. I suggest to use the standard term “underlying cause” instead of “basic cause”.

We accepted the suggest of the reviewer and the term was adjusted by “underlying cause”.

10. When ICD chapters are mentioned, I would suggest to use also the chapter number that would allow to find the results more easily in the tables.

The suggestion of the reviewer was accepted, and we included the chapter number.

11. Acronyms should be explained only once, the first time that are used (examples in line 28, CoD acronym described in the line above).

The acronyms were adjusted throughout the manuscript, according to the guidelines requested by Plos One Journal.

12. Ln 32-33. The sentence is not clear, what the author mean by “chart review” and “those who knew the medical history”?

The information was taken from medical records and when necessary, interviews were conducted to relatives or witnesses. The clarification was made in the abstract (Ln 27-29)

The terms are clear in the paper but they should be clear also in the abstract. Should it be “medical documentation” and “interviews to relatives”?

Same as previous answer, we clarified in the abstract that medical records were reviewed and when necessary, interviews to relatives were conducted to determine CoDs. The clarification was made in the abstract (Ln 27-29)

Ln 33: the certificate considered “standard” is the one based on the medical documentation and interviews?

Yes, the gold standard certificate is the one based on medical records. In a few cases the standard certificate was fill-in with information from interviews with family members or witnesses (only in case the information in the medical records was incomplete).

13.Ln 116-117, which variable and which points are plotted in the Bland-Altman diagram?

The Bland-Altman diagram, illustration the absolute difference in underlying CoD between the original and gold standard death certificates. The variable plotted in the Bland-Altman diagram was the comparison of underlying CoD grouped by ICD-10 chapter from both the original and gold standard death certificates. We explained in the “results” section (Ln 221-225).

14.Ln 124-130 and table 1. Is the sample analyzed (776 cases) representative of the total deaths in Colombia (in terms of age and sex)? A sentence regarding this aspect and on the possible distortion due to the loss of some cases from the desired sample would be useful.

Yes, it is representative of the total deaths in Colombia (in terms of age and sex). A nominal loss of 4% in the sample is considered a minimal reduction that did not introduce any distortion in the study findings. We explain it in the “results” section (Ln 154-157).

15.Ln 157-164. Confidence interval for Kappa should be used for comments and only robust values should be highlighted. For instance, results found for chapter “pregnancy childbirth and puerperium” are based only on two cases: it is not possible to evaluate false positives and Kappa is misleading. This should be taken into account.

We agreed with the reviewer and remove this information from the manuscript. We considered that it is not possible to determine the kappa statistic with only two cases of the chapter “pregnancy childbirth and puerperium”.

16.Ln 167: The term “level of occurrence” is not clear. The table represents the Deaths by underlying cause according to the standard and original certificate and comparison of figures deriving from the two certificates.

We agreed with this comment and the title of table 3 was changed to: ¨Underlying CoD (ICD-10) agreement metrics between original and gold standard death certificates. Colombia, 2021¨ in the “results ‘section” (Ln 197-198).

17. Table 3. Last column (Change in cause – specific mortality fraction (%)): if the number of deaths for the original certificate is greater than for the standard, why the sign of the indicator is negative? According to the formula (line 114) I would expect a positive sign (and vice-versa for positive values).

Regarding this comment, we reviewed and founded an error in the formula in table 3. We adjusted it including the last column as % specific mortality fraction (Ln 200-201). In consequence results changed and were explained according to the findings.

18.Ln 171-177: This part presents the results for men and women (is it correct?), but it seems a repetition of the above comment. I suggest to mention that for most causes the results are comparable in the two genders and successively to focus only on chapters where there are differences.

We agreed with the reviewer's suggestion, and therefore the text was adjusted in the “results” section (Ln 203-217) focusing in the differences between genders.

19. Ln 177: Fig 1 is a table, it should be considered table 4.

We adjusted the table 4 in the “results” section (Ln 218-220), according to the guidelines of tables and figures requested by Plos One journal.

20.Ln 182: The term validity in this context is not clear (the description of this table should be similar to table 2 which has similar content).

We agreed with the reviewer and adjusted the table 2 in the “results” section (Ln 176-177). We changed the term validity to: “Cross tabulation of underlying CoD (ICD-10) in original and gold standard death certificates. Colombia, 2021”.

21.Figure 1 (should be table 4): the columns’ titles for women are different than for men and they have different order.

We agreed with the reviewer and the table 4 was adjusted in the “results” section (Ln 218-220). We corrected the men's and women's columns.

22.Ln 207-209: The authors report underestimation for ‘circulatory system disease’ but the results seem to show that the original death certificate overestimates them. The same occurs for ‘diseases of respiratory system’. This term should be used when the original death certificate, which is under evaluation, detects more cases than the standard. The use of the term underestimation considering the false negatives is misleading. I would suggest to use the CSMF, which is calculated but little used in comments.

We agreed with the comment of the reviewer and adjusted in the “discussion” section (Ln 247-255), considering that it was an overestimation of diseases of the respiratory system in the original certificate. We also agreed using the CMSF in the described discussion paragraph.

23.Ln 210: The result about ‘pregnancy, childbirth and puerperium’ is very counterintuitive, since death certificates are often considered to underestimate maternal mortality, while examining additional documentation should allow to detect more accurately the cases. What is the authors’ opinion on this result?

We considered removing this information from the manuscript, because it was not appropriate to determine results with few cases from this chapter, as we explained in comment No.15. Additionally, these cases do not have complete information in the medical records, and it was not possible to determine a precise cause of death.

In addition, it is surprising that there are 7 cases classified in chapter XVII (Symptoms and signs) by the standard certificates which is based on additional documentation. Is there some explanation for these cases?

In Colombia, analyzes of maternal deaths are carried out to conclude whether the death corresponded to a pregnant woman according to the definitions used in public health surveillance. For these seven cases, the sources of information (medical records) were not complete and did not contribute to the causal chain of death. For this reason, the basic cause of death could not be related in chapter XV (pregnancy, childbirth and puerperium).

24.Ln 216-218: Could authors rephrase this sentence? It is not very clear.

Yes, we can rephrase this sentence. We left it as follows: “A substantial proportion of death certificates (79%) were prepared in health services by doctors, in some cases by health professionals with limited experience in this specific task or with a greater workload, which influences in not carefully reviewing the medical records to formulate the CoD sequence.”

We adjusted the sentence in the “discussion” section (Ln 263-266).

25.Ln 224: Are there differences for deaths occurred in hospital compared to other places?

Yes, there are differences between deaths occurred in hospital compared to other places. We added a paragraph explaining this difference with its respective bibliographic reference. We added a paragraph with this subject in the “discussion” section (Ln 262-273).

Are there differences for source of determination of the cause of death? If possible, some comments could be provided in the discussion (even if not provided in the results).

Yes, there are differences between the source of determination of the cause of death, depending on if the death occurs in the hospital or other places. We explained this subject in the “discussion” section (Ln 262-273).

**Major points:**

26. The article essentially argues that death certificates are incomplete and imprecise. The apparent aim of the article is to estimate the accuracy of this information in some way.

Yes, we agreed that the aim of this research is to estimate accuracy. In that way we adjusted the title and aim of the manuscript according with this comment.

In the methods section, it describes how this was achieved, using statistical measures such as kappa and clinical record data for these comparisons.

Yes, we considered changing the term "validity" to "accuracy" in the title and aim of the manuscript, according to the methodological design and the use of kappa statistic.

Moreover, it excessively dwells on situational aspects and also introduces references that appear disjointed, hindering the reader's understanding of whether these are issues pertinent to countries similar to Colombia, or if they represent a standard that should be adopted.

We agreed with the reviewer comment. We reviewed the introduction which was very long and excessively dwells on situational aspects, so it was adjusted to make it more concise and coherent with the aim of the study. The referenced bibliography refers to a statistical calculation used in several studies worldwide and to a topic related to the certification of deaths process that happens in all countries.

27. Informed Consent and Access to Records: One of the significant methodological issues is the lack of clarity around consent for accessing clinical records and comparing them with death registrations. It is not stated whether consent was obtained from the deceased's family or from the physician who completed the death certificate. Clarification on the consent process is crucial to address potential ethical concerns.

In this regard, on April 27, 2022, the Committee on Ethics and Research Methodologies of the National Institute of Health (In Spanish Committee on Ethics and Research Methodologies - CEMIN) stated that “the National Institute of Health through the National Health Observatory , can act as a health authority and within the framework of its missionary functions request from the respective Health Providing Institutions (IPS) access to medical records in accordance with current regulations (Art. 20, Law 57 of 1985; Art. 38 , literal d, Law 23 of 1981; Art. 14, paragraph 3 of Resolution 1995 of 1999).

Then, on July 19, 2022, the same Committee authorized verbal interviews through telephone calls to the relatives or legal representatives of the deceased to complement the missing information in the medical record. It was recommended to “record the telephone call from the beginning, with the prior acceptance of the interlocutor, in such a way that their verbal informed consent was recorded to provide the information required for the exclusive purpose of this activity.”

We adjusted ethics considerations in ´methods ‘section (Ln 139-152) with a clearer explanation of this subject.

28. Legal Implications of Findings: The article does not discuss how it will address legal issues arising from its findings. Given the association of death diagnoses with matters such as life insurance, as mentioned in the document, this oversight could lead to legal disputes involving the families of the subjects and the healthcare professionals involved.

We agreed with this point, and we added an additional paragraph in the manuscript explaining the analysis of the legal implications.

We added this paragraph in “discussion” section (Ln: 273-279), in the following way:

“In terms of legal connotations of death certificates, it is true that the certifier must recognize the potential effects that the logical elaboration of the CoD will have. Using certain CoDs may provide information that could be used to challenge, reduce, or deny life insurance benefits. Additionally, the death certificate is rarely used as the sole source of information for legal or insurance purposes. Nevertheless, to reduce or avoid these situations, it is crucial to obtain correct and accurate information at all times.”

29. Qualifications of Data Reviewers: The educational background of individuals reviewing the clinical histories is not disclosed. Knowing their qualifications is imperative to assess the credibility of the data interpretation.

Yes, we agreed with this comment and added this information in “methods” section (Ln 103-107), including the educational background of health professionals and their qualifications.

30. Data Anonymization and Interviews: There are significant doubts about the anonymization process, especially since family members or caregivers were interviewed. It is unclear if informed consent was obtained for these interviews and whether the participants were made aware of the potential risks.

We agreed that the anonymization process of the study should be clarified in the manuscript, especially in interviews to relatives. Ethics considerations were adjusted in “methods” section (Ln 146-152).

31. Use of Kappa Statistic: The application of the kappa statistic does not inherently validate the findings. The methodological design seems to focus on measuring agreement or discrepancies but does not support the claimed validity of the results.

According with the comment, we changed the tittle and aim of the manuscript because this is a study of concordance and not validity. The concordance study supports the use of the Kappa statistic.

32. Methodological Framework and Statistical Sufficiency: There is no methodological framework explaining why using a kappa statistic is sufficient for the validity mentioned. Further explanation is needed to justify this approach.

According to the previous response, we made explicit the scope of the analysis, therefore, it was specified that it is a concordance study, adjusting the title and aim of the manuscript.

**Minor Concerns:**

33. The definitions of “false positive” and “false negative” are not provided in either the introduction or the methods section, which could lead to ambiguity in interpreting the study's results.

We agreed with this comment and included these definitions in “methods” section (Ln 124-125).
