# Supplementary material for: Accuracy of information on the underlying cause of death: An analysis in Colombia during the COVID-19 pandemic in 2021
Source: PLoS One. 2025 May 20;20(5):e0320466. doi: 10.1371/journal.pone.0320466 (PMC12092014; doi:10.1371/journal.pone.0320466)
Supplement: Attachment — Submitted filename: Response_to_Reviewers_auresp_2.docx [file pone.0320466.s009.docx]

**Response to Reviewers “Accuracy of Information on the Underlying Cause of Death: An Analysis in Colombia during the COVID-19 Pandemic in 2021”**

Thank you very much for the revision of our manuscript. We sincerely appreciate all your valuable comments and suggestions, which helped us to improve the quality of the manuscript. Our responses to the reviewers’ comments are described below in a point-to-point manner in blue text.

*Originality and Context:

1. Please provide a clearer rationale for the study's significance within the existing literature.

Answer (A): We included the study's significance at the end of the introduction section:

Studies evaluating basic CoD selected in accordance with the International Classification of Diseases, 10th Revision (ICD-10) coding rules, comparing certificates prepared by a certifier (original certification) with those by a researcher (gold standard certification) are scarce (17,18). Shortcomings in both completeness and accurately pose challenges to an accurate accounting of deaths. Inaccurate identification of CoD can detrimentally affect the reliability of public health datasets, thereby impeding local and national response strategies. We included in lines 97– 102.

*Methodological Clarity and Ethical Considerations:

2. There are several methodological and ethical considerations that need to be addressed. The temporal scope of mortality records needs clarification:

A: The temporal scope of the revised mortality records corresponds to 2021 as mentioned in both the title and goal of the manuscript. You can see it in lines 105 and 108.

3. along with details on informed consent procedures, mitigation of biases during data review, and anonymization processes. Please ensure transparency and provide clear explanations for these aspects in the Methods section.

A: With respect to the informed consent: CEMIN authorized the review of medical

records without Informed consent, considering the National Institute of Health’s role

as the national health authority. Lines 182 -188.

The mitigation of possible biases during the data review process in the latest version of the manuscript, included: Health professionals, including five doctors and a nurse with postgraduate degrees in epidemiology and public health, collected and analyzed information from medical records. These professionals underwent prior training in the DANE’s... Lines 129-132.

The anonymization process …

“Anonymized mortality databases from DANE were accessed” Line 183 – 185.

“Ensuring ethical standards and maintaining participant confidentiality throughout the research process was paramount, necessitating rigorous anonymization of the data.” Lines 198 – 201.

*Engagement with Data Sources:

4. Clarify the decision to use data from the National Institute of Health (INS) instead of the National Administrative Department of Statistics (DANE).

A: We don’t use INS database for the analysis. The following sentence was included in methods section for clarity: Death data were obtained from individual death certificates consolidated in the DANE’s mortality databases. Lines 115-116.

*Statistical Analysis and Results:

5. Provide a more detailed explanation of the statistical analysis, including the calculation of false positives and negatives and the rationale behind using kappa statistics solely for establishing validity.

A: The following text was included in the new version of the manuscript: The underlying CoDs in the original (prepared by the certifiers) and gold standard (prepared by the researchers) certificates were compared at the ICD-10-chapter level. The observed percentage of agreement between the underlying CoD in both certificates, the percentage of positive concordance (number of cases in which both data sets report a positive result), false positive rate, and false negative rate were calculated. Additionally, the kappa coefficient was computed to measure the degree of agreement beyond chance (25–28). Lines 147 -152.

The formulas used for these calculations are as follows (Lines 155-164):

Table 1. Methods of calculating different indicators of agreement

|  |  | Reviewer underlying cause | |
| --- | --- | --- | --- |
|  |  | Diagnosis X | The others |
| Original underlying cause | Diagnosis X | A | b |
|  | The others | C | d |

Observed percentage of agreement = (a+d)/(a+b+c+d)

False Positive Rate = b/(a+b)

False Negative Rate = c/(c+d)

Kappa Coefficient = (Po – Pe)/(1 – Pe)

Where:

Po (observed percentage of agreement) = (a+d)/(a+b+c+d)

Pe (expected percentage of agreement) = ([a+c]/[a+b+c+d]) * ([a+b]/[a+b+c+d])+([b+d]/[a+b+ c+d) * ([c+d]/[a+b+c+d])

The kappa statistic is a widely used measure for evaluating validity and reliability (28), accounting for occurring by chance between observers of the underlying CoD in the gold standard and the original certificates. Lines 166 – 168.

6. Correct any errors in the presentation of results, such as those regarding circulatory diseases.

A: Errors in the results were corrected.

Table 2, 3 and 4 were previously corrected. Additionally, we did a thorough review

verifying the calculations taking the standard certificate as gold standard, and it was

found that it really overestimates diseases of the circulatory system. Thanks for your

observation. (Table 2, 3, 4) (Line 241-250 and 309-312)

*Representativeness and Underestimation:

7. Substantiate claims about underestimation of causes of death, especially for rare events, and clarify the representativeness within the sample.

A: Underestimation of the CoD, especially for rare events, and the representativeness within the sample were addressed as follows:

The death certificate may have limitations as a source of mortality data when estimating deaths due to rare events because they are not certified. Underreporting of these events may underestimate the true burden of the disease (37). The capacity of ICD-10 to track deaths from rare events is limited, so little is reported in mortality studies (38). Furthermore, they are not recorded because proximal causes of death mask more distal causes of death, training to complete death certificates is insufficient, opinions on how comorbidities affect the final CoD are disparate (37), and lack of access to medical care. For the results of the study to be generalizable, the sample must be representative of the population; however, for rare events, it may be difficult to obtain a representative sample due to the low frequency of these events. Lines 321 – 330.

With few observations, the Kappa value should be interpreted with caution because inherent variability can affect its precision. Although, it can provide valuable information on the agreement between measurement methods. Furthermore, its confidence interval allows us to get an idea of the variability associated with the result (39). Lines 331 – 334.

*Grammar and style review:

8. Please perform a thorough revision of the manuscript to meet PLOS ONE Criteria for Publication.

A: English style was reviewed in entire manuscript.

**Reviewer #4:**

9. The paper is interesting but a paragraph in the Methods section is needed providing the context of the study, specifically addressing the following points of death certification in Colombia:

A: We have included the paragraph, considering the reviewer's suggestions. To continue, the details: Lines 146-164.

10. 1) Who fills the death certificates (this is already reported in Discussion, but a brief anticipation is needed in Methods)

A: Physicians usually fill out the death certificate. Lines 129-131.

11. 2) Which type of death certificate is adopted: exactly the International Death Certificate from the WHO, or an adaption?

A: The death certificate in Colombia, is designed according to international standards, especially in aspects related to the CoD. Lines 108 – 110.

12. 3) In national mortality statistics, how the underlying cause of death is selected: by trained coders, or by a software as in the US (ACME) or in Europe (IRIS)?

A: When available, the automated coding system IRIS was obtained, but for records not coded in IRIS, manual assignment of the ICD-10 code was performed. Lines 112 – 113.

13. 4) Which version of the ICD-10 was adopted in the study period?

A: “In 2022, DANE used the 2019 version of ICD-10”. Lines 111-113.

Moreover, in Methods authors should clearly specify the following:

14. What are they comparing, e.g. the disease on the underlying cause line in the death certificate compiled by the certifier, or the underlying cause of death selected according international coding rules by a coder or by software? This might not be important for the gold standard, since in a “perfect” death certificate the two conditions should coincide, but is relevant for the original death certificate: as an example, in a death certificate reporting pulmonary embolism in part I and cancer in part II, the latter (and not pulmonary embolism) will be selected according to international coding rules as the CoD

A: We are comparing the underlying cause of death selected by the researches according to international coding rules coded in CoDs by an expert nosologist and the underlying cause line of the original death certificate completed by the certifier. Lines 129-148.

15. Authors should explicitly state already in Methods that they are measuring agreement at the chapter level of the ICD-10

A: We have included: “certificates were compared at the ICD-10-chapter level”. Line 148.

16. Results need some improvement: a footnotes in all Tables with an abbreviated description of chapter numbers (e.g. “Infectious” for chapter I). Moreover, in page 11, last lines, an error was left from the previous version (circulatory diseases exhibited higher percentages of false positives, not of false negatives)

A: We made the adjustments indicated in tables 3, 4 and 5 and page 11 according to circulatory diseases (now page 14, lines 265-266).

17. Lastly, authors should carefully check for typos through the manuscript: only as a first example, at the end of Methods in the Abstract, “…. and calculate kappa value were utilized” should be replaced with “… and kappa value were utilized …..”

A: We made the corresponding adjustments through the manuscript.

**Major Points**:

Originality and comparative analysis:

18. The article's novelty is not clearly established. A comparative analysis with previous years or subsequent data (2019, 2020, 2022) would provide context to evaluate changes in accuracy over time, and specially the relation/effect of COVID-19 in registries precision.

A: The novelty of the article is established as follows: Studies evaluating basic CoD selected in accordance with the International Classification of Diseases, 10^th^ Revision (ICD-10) coding rules, comparing certificates prepared by a certifier (original certification) with those by researcher (gold standard certification) are scarce (17,18). Shortcomings in both completeness and accuracy pose significant challenges to accurately accounting for deaths. Inaccurate identification of CoD can detrimentally affect the reliability of public health datasets, impeding both local and national response strategies. Lines 97 – 102.

Methodological clarity and ethical considerations:

19. The temporal scope of the mortality records reviewed remains unclear (It needs to be precised).

A: The temporal scope of the revised mortality records corresponds to 2021. Please see line 105 and 108.

20. Additionally, the article must address whether informed consents were secured for the review of medical records and how potential biases during the data review process were mitigated. It is also critical to clarify the anonymization process in methodology and ethical considerations involved, especially in relation to the conducted interviews (include questions applied to participants in appendices).

A: Informed consent was not required for the review of medical records. The CEMIN authorized the review of the medical records, considering that the National Institute of Health is the health authority in the country. Lines 185 - 188.

- Health professionals, including five doctors and a nurse with postgraduate degrees in epidemiology and public health, collected and analyzed information from medical records. These professionals underwent prior training in the DANE’s... Lines 129 – 131.
- To address gaps in the medical records, researchers contacted the residences of the deceased, aiming to speak with a relative or acquaintance. Researchers identified themselves as representatives of the National Institutes of Health, explained the study’s purpose, and outlined the protocol. Consent was obtained from participants for their involvement in the study and for the audio recording of the conversation. If participants declined, they were thanked, and a follow-up call was made. If audio recording was not permitted, permission was sought to take written notes. Lines 189 – 195.

21. Sampling and data source engagement with DANE: The sampling method from the 326,833 certificates and the decision to utilize data from the INS instead of DANE requires clarification. It's essential to understand whether the co-authors engaged with DANE regarding the study. Notably, the lack of substantial references from DANE or local studies to corroborate the significance of this issue is a concern, with only reference number 13 addressing local context, and references 14 to 18 providing some local insight. A stronger consideration of DANE as the official data source is necessary.

A: We have included the following settings:

- Death data were obtained from individual death certificates consolidated in the DANE’s mortality databases. Lines 115 – 116.
- In relation to the study, none of the co-authors were involved with DANE.

We incorporate references from the few local research as well as references pertaining to DANE. The text included is the below:

- In Colombia, the National Department on Statistics (DANE by its Spanish acronym) is the government entity responsible for official statistics, including vital statistics. In the 1990s, the Civil Registry and Vital Statistics System (SRCEV by its Spanish acronym) was established to organize and standardize information on births and deaths. This system comprises Civil Registry and Vital Statistics subsystems (19). The Civil Registry subsystem registers and stores information about vital events and their characteristics for legal and administrative purposes (20). The Vital Statistics subsystem collects, processes, and disseminates information on all births and deaths that occur in the country, enabling DANE to prepare official statistics of these events (19). In 2019, the “National yearbook of vital statistics Colombia 2019” was published, dedicating a chapter to data quality, excuding the quality of the underlying CoD information (21). Lines 86- 96.

Statistical analysis and methodology:

22. Concerns arise regarding the calculation of false positives and negatives, which need to be clarified (specify whether these measures support the analysis). The methodological rationale behind using kappa statistics solely for establishing validity is insufficiently explained.

A: For the calculation of false positives and negatives:

- The underlying CoDs in the original (prepared by the certifiers) and the gold standard (prepared by the researchers) certificates were compared at the ICD-10-chapter level. The observed percentage of agreement between the underlying CoD in both certificates, the percentage of positive concordance (number of cases in which both data sets report a positive result), false positive rate, and false negative rate were calculated. Additionally, the kappa coefficient was computed to measure the degree of agreement beyond chance (25-28). The 95% confidence intervals (95% CI) for these metrics were also estimated. The formulas used for these calculations are as follows (Lines 147 – 164):

Table 1. Methods of calculating different indicators of agreement


|  |  | Reviewer underlying cause | |
| --- | --- | --- | --- |
|  |  | Diagnosis X | The others |
| Original underlying cause | Diagnosis X | a | B |
|  | The others | c | D |

Observed percentage of agreement = (a+d)/(a+b+c+d)

False Positive Rate = b/(a+b)

False Negative Rate = c/(c+d)

Kappa Coefficient = (Po – Pe)/(1 – Pe)

Where:

Po (observed percentage of agreement) = (a+d)/(a+b+c+d)

Pe (expected percentage of agreement) = ([a+c]/[a+b+c+d]) * ([a+b]/[a+b+c+d])+([b+d]/[a+b+ c+d) * ([c+d]/[a+b+c+d]).

The justification for using kappa statistics methodologically:

- The kappa statistic is a widely used measure for evaluating validity and reliability (28), accounting for occurring by chance between observers of the underlying CoD in the gold standard and the original certificates (29). Lines 166 – 168.

Representativeness and underestimation:

23. The claims of underestimation of certain causes of death and the representativeness of these within the sample need substantiation, especially for rare events (additionally evaluate how to operate kappa in circumstances where there are few observations).

A: The underestimation of certain causes of death and their representativeness within the sample, especially for rare events, is justified as follows: The death certificate may have limitations as a source of mortality data when estimating deaths due to rare events because they are not certified. Underreporting of these events may underestimate the true burden of the disease (37). The capacity of ICD-10 to track deaths from rare events is limited, so little is reported in mortality studies (38). Furthermore, they are not recorded because proximal causes of death mask more distal causes of death, training to complete death certificates is insufficient, opinions on how comorbidities affect the final cause of death are disparate (37), and lack of access to medical care. For the results of the study to be generalizable, the sample must be representative of the population; however, for rare events, it may be difficult to obtain a representative sample due to the low frequency of these events. Lines 321 – 330.

A: Regarding how Kappa operates in circumstances where there are few observations, we include:

With few observations, the Kappa value should be interpreted with caution because inherent variability can affect its precision. With a small number of observations, it can provide valuable information on the agreement between measurement methods. Furthermore, its confidence interval allows us to get an idea of the variability associated with the result (39). Lines 331 – 334.

**Minor points**

24. Definition of "gold standard certificate": The term "gold standard certificate" is used without explanation (could be difficult for reader, use a unique term). A clear definition is necessary to avoid confusion and ensure reader comprehension.

A: We have included the definition of "gold standard certificate": “gold standard (prepared by the researchers) certificates” Lines 147– 148.

Interviews and recording custodianship:

25. The use of telephone interviews requires clarification on consent, custodianship of the recordings, contingency plans for non-participation, and the refusal rates, as well as transparency about the questions asked (include instrument in appendices).

A: Information on informed consent, recording custody, and openness about the topics posed during phone interviews were mentioned in the response to the second question included in the major points.

Inclusion of interview data in results:

26. The application of interview data within the results is not demonstrated, which raises questions about its influence on the study's conclusions.

A: In the results, “Table 2. Characteristics of analyzed deaths” includes information related to the frequency of interviews with family members or witnesses that were carried out to obtain information about the death. The telephone interviews carried out are also reported.

27. Decimals in sample size: The presence of decimals in sample size in Table 1 raises questions about the data presentation, which typically requires whole numbers.

A: Table 2 shows the characteristics of the analyzed deaths. The data that includes decimals corresponds to the mean age at death in years and its standard deviation (SD).

| Mean age at death, in years | 65.9 (SD: 21.4) | 64.4 (SD: 20.9) | 65.1 (SD: 21.1) |
| --- | --- | --- | --- |

Line 214

Discussion of accuracy vs concordance:

28. The conclusions focus on concordance rather than accuracy, which is a central theme. The impact of the pandemic on the study's validity should be assessed, especially since the title starts with COVID-19, implying a primary concern with the pandemic's influence on data precision requiring to analyze additional years.

A: We include in the conclusion:

On the other hand, the general kappa statistic by sex was high for the CoD of infections, digestive diseases, perinatal conditions, congenital anomalies and signs, symptoms, and poorly defined conditions; and almost perfect for neoplasms and COVID-19. Lines: 372-375.

Bibliographical Strength and Relevance:

29. The bibliographic support for key arguments, particularly regarding the relevance of accuracy in death certification to public health decision-making, is insufficient. The introduction would benefit from robust, primary sources, and all references should comply with citation standards, including DOI where applicable.

A: To highlight the relevance of precision in death certification for public health decision-making, the following paragraph has been included with its corresponding bibliographic support:

- The accuracy and quality of the information provided by death certificates cannot be understated, as they serve as a valuable source of public health information (40). The information they provide supports public health authorities in making decisions. In this sense, the continuing education of health personnel, adequate training in the university curriculum and in the workplace, formalized processes for reviewing death certifications, the use of automated systems to record the cause(s) of death, and selecting the underlying CoD can improve accuracy in death certification (8,31,40,41). Lines 377 – 382.
- We have included references related to accuracy in death certification, public health decision-making, and the DOI where it was missing.
