# Supplementary material for: Accuracy of information on the underlying cause of death: An analysis in Colombia during the COVID-19 pandemic in 2021
Source: PLoS One. 2025 May 20;20(5):e0320466. doi: 10.1371/journal.pone.0320466 (PMC12092014; doi:10.1371/journal.pone.0320466)
Supplement: Attachment — Submitted filename: Response to Reviewers R2.docx [file pone.0320466.s011.docx]

Bogotá, D.C, September 17^th^, 2024

Andres Mauricio Acevedo-Melo, M.D.

Academic Editor

PLOS ONE

Dear Editor,

We sincerely thank you and to the reviewers for the valuable feedback on our manuscript titled “**Agreement of Information on the Underlying Cause of Death: An Analysis in Colombia during the COVID-19 Pandemic in 2021**”. PONE-D-23-33548R2. We appreciate the time and effort you have taken to review our work and provide insightful comments. We have carefully considered each of the points raised and have made the necessary revisions to the manuscript. Please find our detailed responses to the reviewers’ comments below. We don’t have changes to your financial disclosure.

**Journal Requirements:**

Please review your reference list to ensure that it is complete and correct. If you have cited papers that have been retracted, please include the rationale for doing so in the manuscript text or remove these references and replace them with relevant current references. Any changes to the reference list should be mentioned in the rebuttal letter that accompanies your revised manuscript. If you need to cite a retracted article, indicate the article’s retracted status in the References list and also include a citation and full reference for the retraction notice.

R: Reviewed, not retracted articles identified.

**Additional Editor Comments:**

Dear Authors,

Thank you for submitting a revised version of your manuscript. Before it can be considered for publication, there are a few issues that need to be addressed. Please review the following main points, which are related to PLOS ONE publication criteria and highlight the concerns raised by the reviewers:

*Conclusions Supported by DatR: Your manuscript uses the terms “accuracy” and “agreement” interchangeably. However, it is important to note that kappa statistic measures agreement, while false positive and negative rates estimate discrimination.

R: We changed the title from ‘accuracy’ to ‘agreement’ and across all the manuscript.

Additionally, the distinction between “original” and “standard” death certificates, role of participating institutions and custodianship need clarification. Please revise these concepts throughout the manuscript to ensure clarity.

R: A clarification was made in methods section: The “original” death certificate is the one issued by the authorized health personnel at the time of death. The “standard” death certificate is the one prepared by the researcher team after checking the available sources of information. (Lines 139 to 141).

In addition, we included now the clear role of the institution: The researchers are part of the staff of the National Health Observatory of the National Institute of Health of Colombia, which is responsible for the custody of the information collected. (Lines 194 to 196).

Also, provide clear descriptions and interpretations of measures such as the Change in Cause-Specific Mortality Fraction (CCSMF):

R: A new paragraph in results section with the interpretation poof findings was included: The CCSMF for Colombia are not significant; they were <10% for some of the major groups of CoDs, such as infectious diseases, cancer, and diseases of the circulatory system. However, CCSMF were high for other disease groups of public health importance, including genitourinary diseases and external causes (overreporting, >10%). In contrast, the group of diseases of the endocrine system showed underreporting (< -10%) (Table 4). In men, CCSMF were evident in genitourinary and external causes (overreporting, >10%) and in infectious and respiratory diseases (underreporting, line 294). Among women, infectious and genitourinary diseases were overreported and endocrine and digestive diseases were underreported (Table 5). (Lines 288 to 296)

and the Bland-Altman diagram axis and legends:

R: A new version of Fig. 1 is included.

*Clarity and Language: The manuscript should be written in clear, standard English. Please conduct a thorough English language review and address the mistakes highlighted by the reviewers.

R. English language was reviewed, and mistakes corrected.

The following typos and issues were also noted: the word “Charts” is used in the abstract submission while “records” is used in the manuscript abstract

R: In the abstract ‘clinical record’ was replaced by ‘medical records’.

a repeated sentence appears on lines 35-38

R: Suppressed. Thanks

offres” should be corrected to “offers” on line 56

R: Corrected. Thanks

and the acronym “IRIS” on line 112 should be explained

R: Adjusted and explained as ‘IRIS’ (automated coding software program that enables medical death certificates to be coded according to ICD standards) in lines 112 to 114.

Please also review and correct subject-verb agreement and coherence issues, particularly between lines 280 and 284.

R: Correspondence was corrected.

**Reviewers' comments:**

Comments to the Author

Reviewer #1: The manuscript needs clarity on data sources, terminology, and the impact of the COVID-19 pandemic on death certificate accuracy.

R:

- Data sources:

Methods section now includes the following: Primary and secondary sources of information were used. The former compiled information from the medical records of the deceased included in the study. The second consisted of the DANE 2021 mortality database, which compiles information from death certificates issued in the country. (Lines 105 to 109).

- Terminology:

Revised and adjusted through the manuscript.

- The impact of the COVID-19 pandemic on death certificate accuracy

In results a new paragraph about COVID-19 impact was included: Of the 776 death certificates analyzed, COVID-19 was considered the underlying CoD in 231 (29.8%) of the "original" and 247 (31.8%) of the "standard" certificates. When these certificates were compared, 212 (27.3%) agreed that COVID-19 was the underlying CoD; agreement between data sources was very good (Kappa 0.84; 95% Cl: 0.79 - 0.88). Of the 19 (8.2%) deaths for which the original certificate indicated that the underlying CoD was not COVID-19, six (2.6%) were due to respiratory diseases other than COVID-19 and five (2.2%) were due to circulatory diseases. Of the 35 (8.2%) deaths for which the standard certificate indicated that the underlying CoD was not COVID-19, seven (2.8%) were due to respiratory diseases other than COVID-19 and 18 (7.3%) were due to circulatory diseases (Table 4). (Lines 255 to 263)

Also, in the discussion we now include: In the other hand, the impact of the COVID-19 pandemic on the accuracy of death certificates could be reflected in the determination of the underlying CoD by circulatory and respiratory diseases other than COVID-19 (underreporting), which were displaced as the underlying CoD. (Lines 373 to 376)

Reviewer #4: Author improved the manuscript. Some minor revision is still needed

1) Abstract: please remove chapter numbers ("circulatory system diseases" is enough for the Abstract, without adding "Chapter IX")

R: Removed the chapter number form the abstract.

2) Careful editing is needed, only as an example Introduction, page 3, row 56: please change "offres" with "offers"

R: English language revision in the new version of the manuscript.

3) Please correct Table 1: under "Reviewer underlying cause", change the right column from "Diagnosis X" to "The others (or better, "All other diagnoses"), and check all the following formulas

R: Adjustment made. Thank you for the suggestion.
