# Supplementary material for: Accuracy of information on the underlying cause of death: An analysis in Colombia during the COVID-19 pandemic in 2021
Source: PLoS One. 2025 May 20;20(5):e0320466. doi: 10.1371/journal.pone.0320466 (PMC12092014; doi:10.1371/journal.pone.0320466)
Supplement: Attachment — Submitted filename: Response_to_Reviewers_auresp_4.docx [file pone.0320466.s012.docx]

**Response to Reviewers**

**Article title: “Accuracy of Information on the Underlying Cause of Death: An Analysis in Colombia during the COVID-19 Pandemic in 2021”**

Thank you very much for the revision of our manuscript. We sincerely appreciate all your valuable comments and suggestions, which helped us to improve the quality of the manuscript. Our responses to the reviewers’ comments are described below in a point-to-point manner in blue text.

Review Comments to the Author

Reviewer #5:

1. ICD chapters encompass a wide range of diseases and conditions. For example, the chapter on "Diseases of the circulatory system" includes various conditions such as myocardial infarction, strokes, and hypertension. Grouping at this level may mask discrepancies in specific causes of death within the chapter.

A/ Agree with the reviewer. To illustrate these discrepancies, and according to your next comment, an analysis was conducted that incorporated the mortality tabulation list 2 (80 causes), to which we added "covid-19", as it was not included at the time the list was developed provided by ICD-10 (lines 159 -160).

2. More detailed categorization, such as using ICD-10 codes at the sub-chapter or individual code level, could provide a clearer picture of the accuracy and quality of cause of death data. It would allow for identifying specific areas where misclassification or inaccuracies are more prevalent. The authors could easily use the mortality tabulation lists (General mortality 1 -103 causes or General mortality 2 – 80 causes) provided in ICD-10 for disease categorisation.

A/ The utilization of ICD-10 mortality tabulation list 2 (80 causes) has facilitated enhanced precision and quality in the basic CoD data. The findings have been integrated into the supplement (Table 1S). Moreover, the results have encompassed " whereas the mortality rate based on ICD-10 mortality tabulation list 2 was 63.4% (492 deaths correct classified) (Table 1S)” (lines 225).

3. Detailed cause of death data is crucial for designing targeted public health interventions. Broad categories may not provide the necessary details to inform specific health policies and resource allocation.

A/ We concur with the reviewer's assessment. In fact, the broad categories fail to provide the requisite level of specificity. Consequently, the concordance rate achieved using the ICD-10 list of chapters was 74%, while with 80 causes list, which contains more detailed information, it was 63.4%.

4. The study acknowledges the limitations of using broad categories and suggests that a more detailed analysis could provide additional insights. However, the broad categorisation might have been chosen for practical reasons, such as data availability or analysis feasibility.

A/ The decision to employ broad categorization was predicated upon pragmatic considerations, with the implementation of a more meticulous examination facilitated by ICD-10 mortality tabulation list 2. Nevertheless, the limited sample size in certain categories precluded the discernment of potential discrepancies. This observation was addressed in the limitations section of the discussion, as follows: " Fourth, the use of broad categories at the ICD-10-chapter level could limit the identification of inaccuracies and the specificity of the analysis. To mitigate this situation, a more detailed analysis was included employing ICD-10 mortality tabulation list 2 to 80 causes plus COVID-19" (lines 382 -385).

5. Future studies could benefit from a more detailed examination of cause of death data using specific ICD-10 codes. This would enhance the understanding of the quality of death certification and help identify specific areas for improvement.

A/ We concur with the reviewer's assertion that future studies should reevaluate the criteria for determining the sample size. This reevaluation is necessary to facilitate a more comprehensive examination of the information pertaining to the causes of death. Such an examination will provide a more profound understanding of the quality of the certification of deaths, thereby enabling the formulation of strategies to enhance it.

In summary, while the broad categorization at the ICD chapter level provides a general overview of the agreement between original and gold standard death certificates, a more detailed analysis would offer greater specificity and potentially more actionable insights.

A/ We agree. It is imperative to acknowledge the value of a comprehensive categorization, which entails the utilization of mortality lists that offer enhanced specificity or the consideration of specific etiological agents, such as cancer. An analysis that is more precise could be conducted.

Another issue is that he chapter-level broad categories of causes of death could include garbage codes (unusable codes for public health decision-making) of medium impact. These are known as ‘Level 3 (medium)’ garbage codes, meaning codes with important implications: causes for which the true underlying cause of death is likely to be within the same ICD chapter. For instance, ‘unspecified cancer’ still provides enough information to know if the UCOD was due to cancer. However, knowledge about the site of cancer is important for public health policy since different strategies are applied for different types (sites) of cancer (i.e. breast versus lung cancer).

The categorization of garbage codes into levels, particularly Level 3 garbage codes, is a useful approach to understanding the quality of cause of death data. Level 3 garbage codes refer to causes of death where the actual underlying cause is likely within the same ICD chapter.

In summary, since the study's use of broad categories aligns with the concept of Level 3 garbage codes, it still needs to be revised in terms of specificity and potential misclassification. Recognizing and addressing the presence of Level 3 garbage codes can help improve the quality of cause-of-death data and enhance the utility of mortality statistics for public health.

A/: We agree with the reviewer about the importance of the garbage codes. We do not include them in the study, but for level 3, they were found only in 22 (2.84%) of the basic CoD certifications. Of these, 14 corresponded to "unspecified cancer".

While the study methodology is generally sound, there are a few potential issues and limitations that could be addressed:

1. As previously mentioned, grouping causes of death at the ICD chapter level is quite broad and may mask specific discrepancies within those chapters. More granular categorization could provide a clearer picture of the accuracy and quality of cause of death data.

A/ As we mentioned, in the new version, we implemented ICD-10 mortality tabulation list 2 (80 causes to which we added COVID-19 at the end of the causes because it was not included at the time the list was compiled). The results are included in the supplement (Table 1S), as we mentioned in response to comments # 1 and 2.

2. Determining the gold standard cause of death relied heavily on medical records and, when necessary, interviews with relatives or witnesses. Medical records may not always be complete or accurate, and interviews can introduce recall bias.

A/ In the discussion section we have include this as a limitation: “Second, in deaths where the clinical documentation was incomplete or in the event of a death that occurred at home, relatives or acquaintances of the deceased were interviewed, presenting possible recall bias” (lines 387 – 390).

3. Although the health professionals were trained and supervised, there is still a risk of observer bias in interpreting medical records and assigning causes of death.

A/ In the discussion section we have included this as a limitation: “Third, potential observer bias in the health professionals who analyzed the medical records, due to the subjectivity. However, the risk of bias is low because the health professionals who analyzed the medical records were trained and were under permanent supervision in the information collection process” (lines 379 – 382).

4. The study used data from the DANE 2021 mortality database and medical records. It did not include other potential sources of information, such as autopsy reports, which could provide additional accuracy in determining the cause of death.

A/ In Colombia, autopsies are very rarely performed for deaths from natural causes. In contrast, autopsies are performed for deaths from external causes, which were excluded from this analysis.

5. The study focused on 92 municipalities, which may only partially represent part of the country. Geographical and demographic variability in the quality of death certification could exist, but this is not captured in this sample.

A/ The study used a probability sample to reach population representativeness. The study universe considered the total 33 territorial entities of the political and administrative division of Colombia (32 departments and the district of Bogotá). Inclusion and exclusion criteria were established that allowed the selection of 92 municipalities out of 770 through random sampling based on computer-generated random numbers. The clarifications were included in the sampling section (lines 124 - 134).

6. The study was conducted during the COVID-19 pandemic, which could have affected the accuracy of death certification due to the high volume of deaths and the strain on healthcare systems. This context should be considered when interpreting the results.

A/ According to this comment, the revised version of the manuscript new consider the context interpretate the results and this is included in the first paragraph of the discussion (lines 309 - 312).

7. While the study received ethical approval and took measures to anonymise data, obtaining consent from relatives or witnesses for interviews could introduce ethical concerns, especially in sensitive or traumatic deaths.

A/ We agree; it is fortunate that this situation did not arise during the data collection process.

8. The findings may not be generalizable to other countries or regions with different healthcare systems, death certification processes, and levels of training for health professionals.

R/ Despite the limitations in the generalizability of the results to other countries or regions, as previously discussed, there are salient elements that merit consideration for the discussion in other contexts. For instance, an evaluation of the quality of information pertaining to the CoD, with a particular focus on the underlying cause as captured by mortality statistics. Additionally, an analysis of the impact of the pandemic on the certification of mortality, the entities responsible for this certification, and the completion of death certificates themselves, among other pertinent aspects.

9. Although the study mentions that health professionals were trained and supervised, it needs to provide detailed information on the extent and quality of this training. Inadequate training could affect the reliability of the gold standard in determining the cause of death.

A/ It is worth mentioning that the training was given by an expert in vital statistics from the DANE, who dealt with the correct preparation of a death certificate, emphasizing the certification of CoD and their correct sequence. During this training, various exercises were carried out to determine the scope and quality of this activity (lines 138 -140).

10. While the sample size is substantial, the study does not discuss potential selection bias in the choice of municipalities and deaths included in the sample. Selection bias could affect the representativeness of the findings.

A/ As previously indicated, the probability sample was obtained through the implementation of random sampling, with a total of 92 municipalities selected based on computer-generated random numbers. This methodological approach was adopted to mitigate the potential for selection bias. The adjustments pertinent to the sampling process are delineated in the following section (lines 126 through 134).

Addressing these issues in future research could enhance the robustness and reliability of the findings and provide more detailed insights into the quality of cause of death data.

A/ The aforementioned aspects were addressed to the greatest extent possible in this analysis. It is acknowledged that future research will necessitate the acquisition of more detailed information regarding the quality of CoD information.

Reviewer #6:

This article aims to estimate the agreement of the underlying Cause of Death in the original death certificate, compared with a gold standard certificate based on information from medical records and relatives, in Colombia during 2021. The authors have used summary measures of agreement such as the proportion of agreement, Kappa, CCSMF and graphical methods such as the Bland-Altman plot. While commending their efforts, the following comments are given with the hope that these will benefit the authors.

1. Please elaborate on the two-stage sampling method a bit more. As an example, it is mentioned that in the second stage, 92 municipalities were selected. It would be better to add that these 92 were selected out of how many. Also, what method was used in this random sampling (i.e. whether done by computer-generated random numbers etc.)? Furthermore, it is mentioned that 33 territorial entities were “considered” in the first stage. It would be nicer to elaborate a bit more on how this consideration was done.

A/ Now sampling section was rephrased to provide additional details. “In the first stage, it was considered conglomerates comprising 33 territorial entities of country’s administrative segmentation (32 departments and the district of Bogotá). In the second stage, from 770 municipalities, were 92 selected using random sampling with computer-generated random numbers, stratified by age groups (<60 years and ≥60 years)” (lines 124-126).

2. Please give a reference to the sample size calculation formula/software that was used.

A/ It was now included: The program EPIDAT 4.2 was used for the random selection of the sample (27). The EPIDAT program, or Epidemiological Data Analysis, is a software program designed for the analysis of epidemiological data. It is currently in its fourth iteration, with version 4.2 released in July 2016 (lines 133-134).

3. In the sample size calculation, please define what variable was taken as “expected proportion of 70%” (i.e. whether it is the expected proportion of agreement?). Was this figure selected based on some previous literature or by some other method?

A/ Now a inclusion was made to give the adequate explanation: “The minimum expected ratio is p, this varies according to the health system, local practices and the method used. It is reasonable to assume that, under appropriate conditions of quality and training in medical certification, the 70% ratio can be considered representative and feasible in systems that prioritize accuracy and consistency in mortality records (23–26)” (lines 130 - 134).

4. Please add any rationale for choosing the design effect of 2.5

A/ An inclusion was made about this: “and an effect of 2.5 (due to heterogeneity on the municipal mortality concentration)” (line 128).

5. It is mentioned that “In cases where medical 143 records lacked sufficient information, interviews with relatives, acquaintances, or witnesses 144 were conducted". Please add a bit more details on who conducted these interviews, in what settings and what kind of information were asked from the respondent etc.

A/ An inclusion was made to attend this comment: “Health professionals conducted telephone interviews with relatives or friends of the deceased in cases where the clinical history was not available, or the information recorded was insufficient to determine the CoD. During these interviews, they were asked about the events that occurred during the illness or the situation that led to the person's death” (lines 146-150).
